# Mucosal Biofilms Are an Endoscopic Feature of Irritable Bowel Syndrome and Ulcerative Colitis

**DOI:** 10.1053/j.gastro.2021.06.024

**Published:** 2021-10

**Authors:** Maximilian Baumgartner, Michaela Lang, Hunter Holley, Daniel Crepaz, Bela Hausmann, Petra Pjevac, Doris Moser, Felix Haller, Fabian Hof, Andrea Beer, Elisabeth Orgler, Adrian Frick, Vineeta Khare, Rayko Evstatiev, Susanne Strohmaier, Christian Primas, Werner Dolak, Thomas Köcher, Kristaps Klavins, Timo Rath, Markus F. Neurath, David Berry, Athanasios Makristathis, Markus Muttenthaler, Christoph Gasche

**Affiliations:** 1 Division of Gastroenterology and Hepatology, Department of Internal Medicine 3, Medical University of Vienna, Vienna, Austria; 2 Centre for Microbiology and Environmental Systems Science, Department of Microbiology and Ecosystem Science, Division of Microbial Ecology, University of Vienna, Vienna, Austria; 3 Joint Microbiome Facility of the Medical University of Vienna and the University of Vienna, Vienna, Austria; 4 Division of Microbiology, Department of Laboratory Medicine, Medical University of Vienna, Vienna, Austria; 5 Department of Cranio-Maxillofacial and Oral Surgery, Medical University of Vienna, Vienna, Austria; 6 Department of Pathology, Medical University of Vienna, Vienna, Austria; 7 Center for Public Health, Department of Epidemiology, Medical University of Vienna, Vienna, Austria; 8 Vienna Biocenter Core Facilities, Vienna, Austria; 9 CeMM Research Center for Molecular Medicine of the Austrian Academy of Sciences, Vienna, Austria; 10 Ludwig Demling Endoscopy Center of Excellence, Division of Gastroenterology, Friedrich-Alexander-University, Erlangen, Germany; 11 Faculty of Chemistry, Institute of Biological Chemistry, University of Vienna, Vienna, Austria; 12 Institute for Molecular Bioscience, The University of Queensland, Brisbane, Australia; 13 Loha for Life, Center for Gastroenterlogy and Iron Deficiency, Vienna, Austria

**Keywords:** Endoscopy, Microbiota, Functional Gastrointestinal Disorders, Bacterial–Epithelial Interaction, ASV, amplicon sequencing variant, BA, bile acid, BF^–^, biofilm negative, BF^+^, biofilm-positive, DAPI, 4′,6-diamidino-2-phenylindole, GI, gastrointestinal, IBD, inflammatory bowel disease, IBS, irritable bowel syndrome, OTU, operational taxonomic unit, PAS, periodic acid-Schiff, PEG, polyethylene glycol, rRNA, ribosomal RNA, SEM, scanning electron microscopy, UC, ulcerative colitis, UCDA, ursodeoxycholic acid

## Abstract

**Background & Aims:**

Irritable bowel syndrome (IBS) and inflammatory bowel diseases result in a substantial reduction in quality of life and a considerable socioeconomic impact. In IBS, diagnosis and treatment options are limited, but evidence for involvement of the gut microbiome in disease pathophysiology is emerging. Here we analyzed the prevalence of endoscopically visible mucosal biofilms in gastrointestinal disease and associated changes in microbiome composition and metabolism.

**Methods:**

The presence of mucosal biofilms was assessed in 1426 patients at 2 European university-based endoscopy centers. One-hundred and seventeen patients were selected for in-depth molecular and microscopic analysis using 16S ribosomal RNA gene amplicon-sequencing of colonic biopsies and fecal samples, confocal microscopy with deep learning–based image analysis, scanning electron microscopy, metabolomics, and in vitro biofilm formation assays.

**Results:**

Biofilms were present in 57% of patients with IBS and 34% of patients with ulcerative colitis compared with 6% of controls (*P* < .001). These yellow-green adherent layers of the ileum and right-sided colon were microscopically confirmed to be dense bacterial biofilms. 16S-sequencing links the presence of biofilms to a dysbiotic gut microbiome, including overgrowth of *Escherichia coli* and *Ruminococcus gnavus*. *R. gnavus* isolates cultivated from patient biofilms also formed biofilms in vitro. Metabolomic analysis found an accumulation of bile acids within biofilms that correlated with fecal bile acid excretion, linking this phenotype with a mechanism of diarrhea.

**Conclusions:**

The presence of mucosal biofilms is an endoscopic feature in a subgroup of IBS and ulcerative colitis with disrupted bile acid metabolism and bacterial dysbiosis. They provide novel insight into the pathophysiology of IBS and ulcerative colitis, illustrating that biofilm can be seen as a tipping point in the development of dysbiosis and disease.

## Graphical abstract

**Figure:**
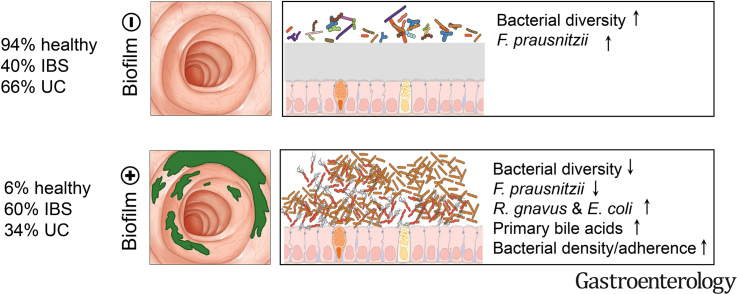

## Teaser

Bacterial biofilms were observed by colonoscopy as yellow-green membranous layers on the mucosa of the small and large intestinal junction and are specifically prevalent in irritable bowel syndrome and inflammatory bowel disease.

**What You Need to Know**

**Background and Context**

IBS is the most common digestive disorder, affecting up to 15% of the Western population. Involvement of the microbiome in disease pathogenesis has been suggested, as fecal microbiota transplantation leads to symptom improvement.

**New Findings**

Previously unrecognized endoscopically visible biofilms are attached to the mucosa of the ileum and right colon in almost two-thirds of patients with IBS and one-third of patients with UC. They are associated with dysbiosis (ie, overgrowth of *Escherichia coli* and *Ruminococcus gnavus*) and increased fecal bile acid excretion.

**Limitations**

This is the first report on such biofilms observed by colonoscopy from 2 tertiary university-based teaching hospitals. To this point, mechanistic studies on the pathogenicity of such biofilms in gastrointestinal homeostasis are limited. Interventional studies on disruption of biofilms are needed to establish a causative involvement in IBS.

**Impact**

As these biofilms are associated with alterations of microbiota and bile acid metabolism, they may be involved in disease pathogenesis. For the clinician, visualization of biofilms by colonoscopy may provide a new diagnostic characteristic of IBS and disruption of such biofilms may offer a novel treatment path.

Irritable bowel syndrome (IBS) and inflammatory bowel diseases (IBDs) affect 10%–15% and 0.5%–1% of the Western population, respectively, with the prevalence of both increasing worldwide.^1^^,^^2^ Patients with IBS have recurrent abdominal pain and changes in stool habits, but lack obvious signs of gastrointestinal (GI) inflammation. Ulcerative colitis (UC) and Crohn’s disease are the most prevalent forms of IBD and are characterized by a prolonged, debilitating inflammation of the GI tract, leading to abdominal pain, diarrhea, intestinal blood loss, and anemia. Such symptoms are associated with a substantial reduction in quality of life, as well as a considerable socioeconomic impact with high hospitalization costs.^3^ Although IBDs are diagnosed by endoscopy, no such immediate diagnostic test exists for IBS. Many patients with IBS are disappointed with current symptomatic medical care and lack of a causative treatment approach.^4^ Western lifestyle, including frequent antibiotic therapy and microbiota-altering food additives, have been implicated in disease development.^5^^,^^6^ Recently, alterations in bacterial bile acid (BA) metabolism have come into focus in IBS pathophysiology.^7^^,^^8^ Transplantation of fecal matter from healthy donors leads to a transient improvement of IBS symptoms.^9^^,^^10^ Changes in the relative abundance of bacterial taxa have been observed via high-throughput sequencing,^11^, ^12^, ^13^ but research on bacterial biomass or the spatial distribution of bacterial communities remains limited.

Biofilm formation is a distinct microbial mode of growth in which adherent prokaryotic communities embed themselves in a complex extracellular matrix to obtain competitive advantages. Biofilm-forming bacteria predominate numerically and metabolically in virtually all ecosystems, and are also involved in chronic bacterial infections of the human body.^14^^,^^15^ While in a healthy gut bacterial growth is usually scattered as small microcolonies,^16^^,^^17^ polymicrobial biofilms have been observed microscopically in IBD, GI infections, right-colonic cancer, and familial adenomatous polyposis.^18^, ^19^, ^20^, ^21^, ^22^ However, a macroscopically visible aspect of biofilm formation in the intestine has never been considered. Stressors on the microbiota, such as overactivation of the immune system in IBDs,^23^ chronic use of microbiome-altering pharmaceuticals^24^ (including immunosuppressive medication, proton pump inhibitors, or recurrent use of antibiotics), and food additives (eg, with antimicrobial and/or detergent activity), as well as GI infections and excessive hygiene,^25^ lead to selection pressures that might trigger microbial defense mechanisms, such as orchestrated biofilm formation.^26^

In this work, we systematically studied 2 endoscopy cohorts with a total of 1426 patients, demonstrating that regularly observed yellow-green adherent layers of the ileum and right-sided colon are indeed biofilms that are readily visible during high-definition white light endoscopy. Such biofilms are highly prevalent in IBS, to a lesser extent in IBDs, and in a post-organ transplantation cohort. We further applied a range of multidisciplinary techniques including 16S ribosomal RNA (rRNA) gene amplicon sequencing, scanning electron microscopy (SEM), confocal microscopy with deep learning–based image analysis, in vitro biofilm formation assays, and metabolomics to characterize these biofilms. We thereby provide advanced understanding of their origin and novel opportunities for future diagnosis and treatment options.

## Materials and Methods

### Screening for Endoscopically Visible Biofilms

The presence of endoscopically visible biofilms was assessed in an international multicenter trial at the Vienna General Hospital, Austria (n = 976) and at the University Hospital Erlangen, Germany (n = 450), for a total of 1426 patients. Endoscopically visible biofilms were defined as an adherent layer on the intestinal surface, despite polyethylene glycol (PEG) –based bowel preparation, which either resist detachment by jet washing or detach in a film-like manner (Supplementary Video 1). Bowel preparation of each patient was scored using the Boston Bowel Preparation Scale.^27^ We excluded patients with a Boston Bowel Preparation Scale score <6 from the analysis to minimize the possibility of false-positive cases. As the type of bowel preparation might have an influence on the appearance of intestinal biofilms, we standardized our cohort by excluding all patients that had non–PEG-based preparations and all patients had a standardized bowel preparation regimen (ie, high-volume PEG and appointment at the next day between 8 am and 1 pm). If the cecum was not reached during endoscopy, patients were also excluded. After applying our exclusion criteria, 1112 patients were analyzed (756 from Austria and 356 from Germany). Multivariate logistic regression with biofilm status as a dependent variable and disease cohort and country as independent variables was used to assess association of disease cohorts with endoscopically visible intestinal biofilms. Calculations were performed using R.^28^

### Sample Collection

One hundred and seventeen patients (56 with IBS, 25 with UC, and 36 controls undergoing colorectal cancer screening with normal findings at colonoscopy) from the Vienna cohort were selected for in-depth molecular and microscopic analysis. Samples were collected during colonoscopy and processed immediately. Biofilm-positive (BF^+^) biopsies were taken from an area with an endoscopically visible biofilm (cecum or ascending colon). Additional biopsies were taken from BF^+^ individuals at least 10 cm distal from the biofilm area (Distal-Bx; see Supplementary Figure 4*I* for a depiction of sampling sites). Biopsies from biofilm-negative (BF^–^) patients were also taken from the cecum or ascending colon.

### Microscopic Analysis of Mucosal Biofilms

Colonic biopsies were analyzed by SEM, confocal microscopy, and bright-field microscopy. To quantify bacterial densities, total number of bacteria and presence of adherent bacteria, we trained U-Net, a recently published deep learning algorithm,^29^ on confocal microscopy images of 4′,6-diamidino-2-phenylindole (DAPI)–stained biopsy sections. An unmodified exemplary picture of detected bacteria (that was not part of the training set) is presented in Supplementary Figure 2*A*. Confocal microscopy images were obtained of all areas with visible bacteria per section. Bacterial density and adherent bacteria density were determined as the maximum number of bacteria in a 144.72 × 144.72 μm image. The total number of bacteria and adherent (within 3 μm of the epithelium) bacteria were calculated as the sum of all images for each biopsy and normalized to the epithelium length (to adjust for the size of biopsy sections, as determined on neighboring H&E-stained sections). Thickness of methacarn-fixed surface layer was assessed with bright-field microscopy of H&E and periodic acid-Schiff (PAS)–stained sections. For each biopsy sample, a whole biopsy section was analyzed. The trained U-Net model and data to replicate Supplementary Figure 2*A* are publicly available at GitHub. For a more detailed description of the microscopic analysis, PAS staining, fluorescence in situ hybridization, and sample numbers, see Supplementary Methods and Supplementary Figure 3.

### Molecular Analysis of Mucosal Biofilms

DNA of colonic biopsies and stool samples was extracted using the standard QIAamp DNA stool mini kit protocol (Qiagen) modified by an initial bead-beating-step with Lysing Matrix E tubes (MP Biomedicals) and a Precellys 24 homogenizer (Bertin instruments) with 5200 rpm 3 × 30 seconds for colonic biopsies and 5500 rpm 1 × 30 seconds for stool samples. Bacterial 16S rRNA gene copy number was quantified using quantitative polymerase chain reaction and normalized to the total amount of double-stranded DNA (assessed with a Quant-iT PicoGreen dsDNA Assay Kit). Metabolomics was performed on a subset of ileal biopsies (5 BF^+^ biopsies and 5 BF^–^ biopsies) using liquid chromatography–mass spectrometry

(Orbitrap Fusion Lumos Tribrid; Thermo Fisher Scientific) for lipid analysis and liquid chromatography–tandem mass spectrometry (6470 triple quadrupole; Agilent Technologies) for metabolite analysis. BA composition of stool samples was analyzed using liquid chromatography–tandem mass spectrometry (TSQ Quantiva; Thermo Fisher Scientific). Methodology, sample numbers, and bioinformatics workflow are described in more detail in the Supplementary Methods and Supplementary Figure 3.

### Analysis of Bacterial Community Composition and In Vitro Biofilm Formation Assay

Colonic biopsies and stool samples were subjected to 16S rRNA gene amplicon sequencing using Illumina MiSeq technology and an established pipeline.^30^ Amplicon sequence variants (ASVs) were inferred with the DADA2 R package,^31^ taxonomic classification was performed with SINA, version 1.6.1.^32^ Differences in bacterial community composition and correlations to BA data were analyzed with DESeq2^33^ and Rhea scripts.^34^ Patient characteristics and sample size, used for 16S rRNA gene amplicon analysis, are displayed in Supplementary Table 5 and Supplementary Figure 3. In vitro biofilm formation experiments were done with bacterial strains isolated from biofilm specimens using an established microtiter plate biofilm assay (for details see Supplementary Methods).^35^

### Data and Code Availability

16S rRNA gene amplicon sequencing data was deposited under the BioProject accession number PRJNA644520. The trained U-Net model for bacteria detection in confocal fluorescence microscopy images of DAPI-stained sections of human intestinal biopsies was deposited at GitHub, including a short tutorial on how to apply it to similar projects and to reproduce Supplementary Figure 2*A* (github.com/MaximilianBaumgartner/U_Net_bacteria_detection).

### Ethics Statement

The study was reviewed and approved by the ethics committee at each study site: Medical University of Vienna (EK-Nr: 1617/2014, 1780/2019, 1910/2019), University Clinic Erlangen (264_19 B). All study participants gave written informed consent before providing samples. The study was conducted in accordance with the ethical principles expressed in the Declaration of Helsinki and the requirements of applicable federal regulations.

## Results

### Endoscopically Visible Bacterial Biofilms Are Present in the Ileum and Colon

**Figure 1:**
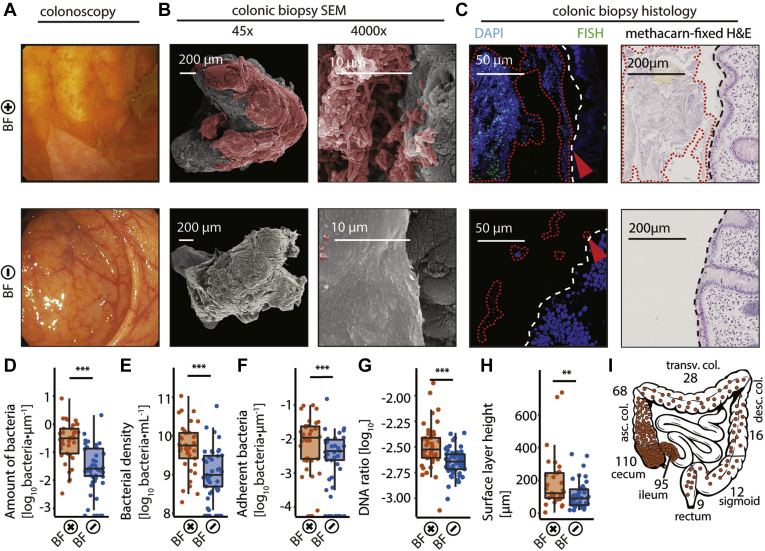
Endoscopically visible biofilms consist of dense bacterial agglomerations. (*A–C*) Comparison of representative endoscopic pictures and biopsies of patients with (BF^+^ IBS, *top panel*) and without (BF^–^ control, *bottom pane*l) macroscopically visible biofilms. (*A*) *Endoscopic picture* of a yellow-green layer adhering to the intestinal mucosal surface of a BF^+^ patient, which is not present in BF^–^ patients. (*B*) SEM of the same patients, confirming the presence of tightly packed bacteria adhering to the epithelium in BF^+^ biopsies (bacteria in *red*). BF^–^ biopsies had an intact mucus layer with foci of scattered bacteria. (*C*) Biopsy sections stained with DAPI (*blue*) and fluorescence in situ hybridization with a general bacteria probe Mix EUB338 I-III (*green*) revealed densely packed bacteria in direct contact with the epithelium (*red arrow*) in BF^+^ biopsies, compared with scattered bacteria distant from the epithelium in BF^–^ biopsies. *Dashed white line* marks the border of the epithelium. H&E staining revealed a surface layer comprising mucus and bacteria in BF^+^ biopsies. (*D*) Total number of bacteria normalized to length of epithelium per section (BF^+^ biopsies, *orange*; BF^–^ biopsies, *blue*). (*E*) Maximum density of bacteria in one 144.7 × 144.7 μm confocal microscopy image per section. (*F*) Number of bacteria within 3-μm distance from the epithelium, normalized to length of epithelium per section. (*G*) Ratio of 16S rRNA gene copies to total double-stranded DNA per biopsy. (*H*) Maximum height of methacarn-fixed H&E-stained surface layer on top of the epithelium, per section. (*i*) Location and number of biofilms of the primary study cohort. Most biofilms were observed in the ileum, cecum, and ascending colon. (*D, E, F*) Zero values are displayed on the x-axis, as they are not defined on a log-scale. Statistical analysis: (*D, E, F, H*) Mann-Whitney U test, n = 37 BF^+^, n = 47 BF^–^, (*G*) *t* test on log-transformed data, n = 42 BF^+^, n = 56 BF^–^; ∗∗*P* ≤ .01, ∗∗∗*P* ≤ .001.

**Supplementary Figure 1:**
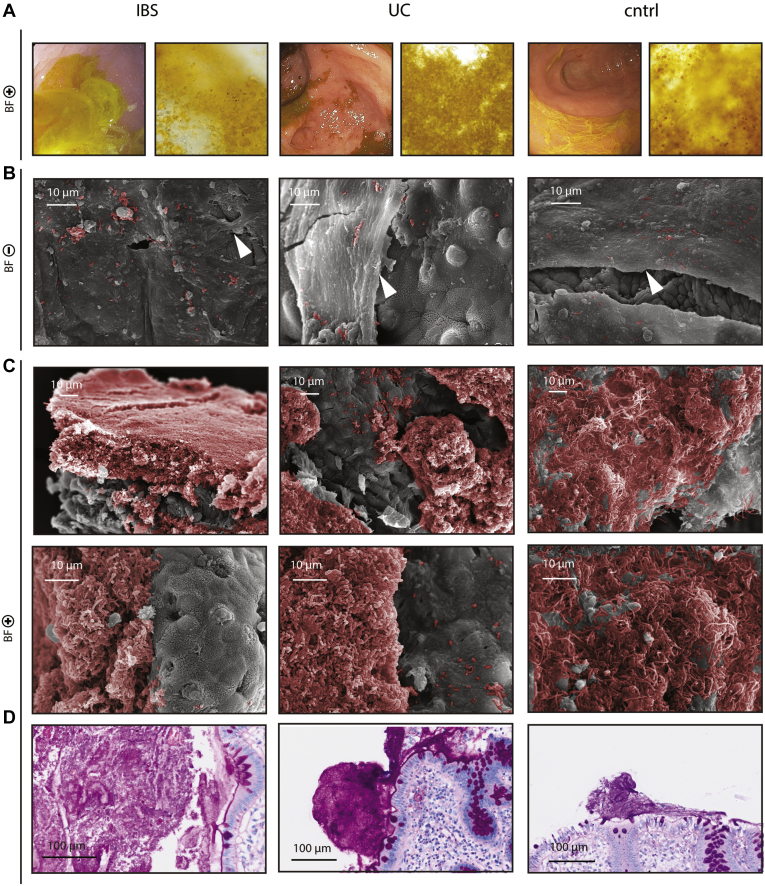
Representative images of biofilm-mode of bacterial growth in patients with IBS, patients with UC, and controls. (*A*) Biofilm flush specimen show yellow color, bacteria, and shed epithelial cells under light microscopy. (*B*) SEM of BF^–^ biopsies shows an intact mucus layer (*white arrows*) with scattered bacteria on top (bacteria color enhanced, *red*). (*C*) SEM analysis of BF^+^ biopsies shows a thick layer of bacterial biofilm in direct contact with the epithelium (bacteria color enhanced, *red*). (*D*) PAS staining of BF^+^ biopsies.

**Supplementary Figure 2:**
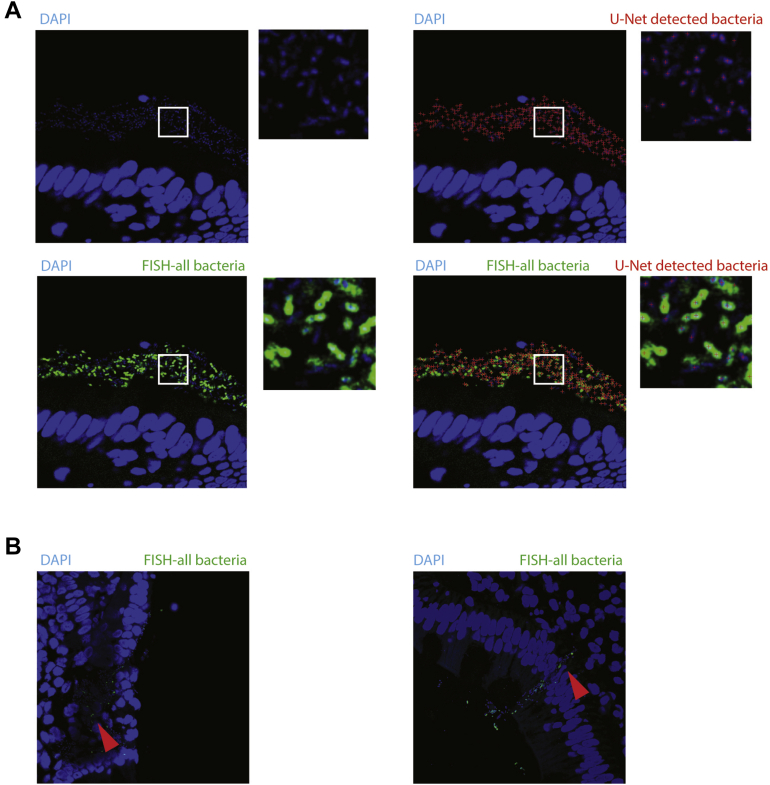
Machine learning–based bacteria quantification and invasion of epithelium in 2 cases of IBS biofilms. (*A*) Unmodified exemplary picture of bacteria detected with U-Net machine learning on an image (colonic biopsy of BF^+^ patient with IBS), which was not part of the U-Net training set (*red crosses*), DAPI (*blue*), and overlay of FISH general bacterial probe signal (*green*). (*B*) Bacterial invasion into the epithelium in 2 cases of IBS biofilms DAPI (*blue*) FISH general bacterial probe (*green*).

**Supplementary Figure 3:**
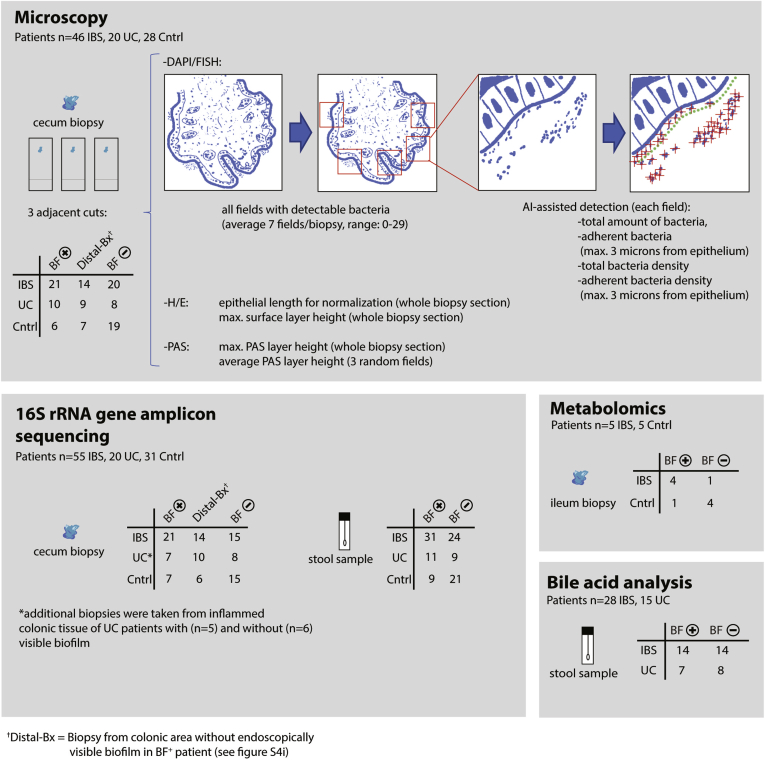
Description of in-depth molecular and microscopic analysis in a subset of the primary cohort. Depiction of in-depth microscopic and molecular analysis, including sample material, patient cohorts, and sample number. Microscopic analysis (*top panel*): analysis was performed on methacarn-fixed cecal biopsies (endoscopically BF^+^, BF^–^, and areas without biofilm from BF^+^ patients [Distal-Bx]), 3 adjacent sections from each biopsy were processed for DAPI/FISH, H&E, and PAS analysis. Confocal microscopy images were obtained from all areas of the whole section with visible bacteria in the DAPI channel. Each image was subjected to artificial intelligence–assisted quantification of bacteria to calculate total number of bacteria normalized to length of epithelium per section (amount of bacteria); maximum density of bacteria in one 144.7 × 144.7 μm confocal microscopy image per section (bacterial density); number of bacteria 3 μm from the epithelium, normalized to length of epithelium per section (adherent bacteria); and maximum density of bacteria up to 3 μm distal from the epithelium in one 144 × 144 μm confocal microscopy image per section (adherent bacteria density). H&E-stained sections were analyzed for the whole length of the epithelium (for normalization) and maximum width of H&E surface layer on top of epithelium per section (surface layer). PAS-stained sections were analyzed for average and maximum width of PAS-stained surface layer on top of epithelium per section (average PAS layer height, maximum PAS layer height). 16S rRNA gene amplicon sequencing (*bottom left panel*) was performed from snap-frozen cecal biopsies (BF^+^ and BF^–^ and areas without biofilm from BF^+^ patients [Distal-Bx]) and stool samples (BF^+^ and BF^–^). Metabolomics (*bottom right panel*) was performed on snap-frozen ileal biopsies (BF^+^ and BF^–^). BA analysis (*bottom right panel*) was performed on stool samples (BF^+^ and BF^–^).

**Supplementary Figure 4:**
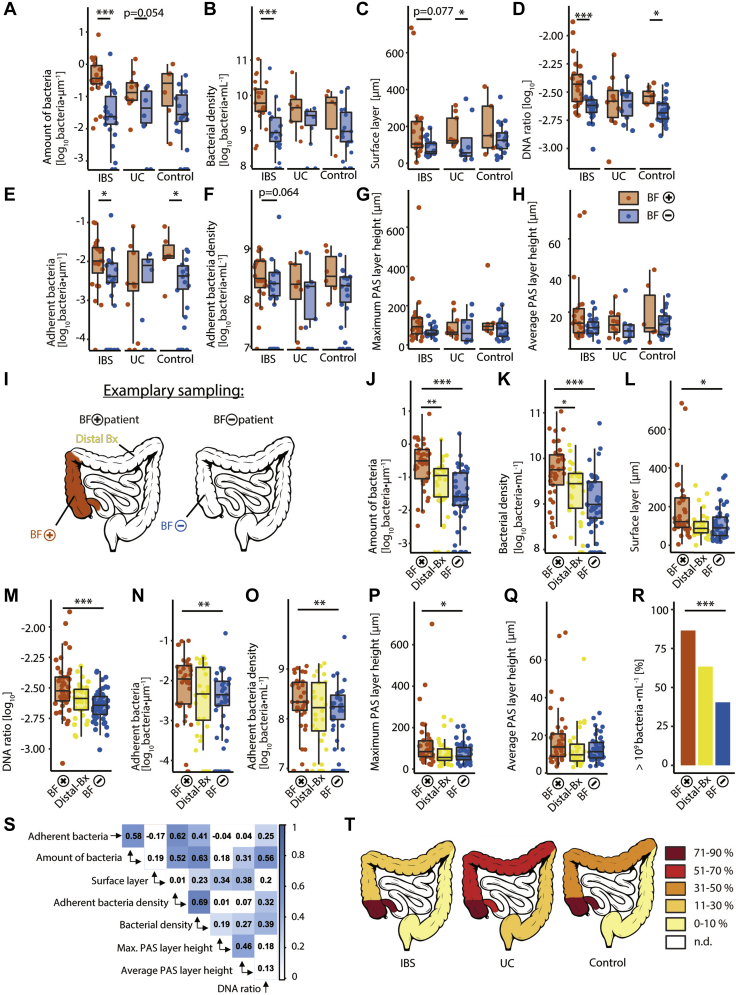
Endoscopically visible biofilms consist of dense bacterial agglomerations independent of disease. (*A–H*) Comparison between BF^+^ biopsies (*orange*) and BF^–^ biopsies (*blue*), separated for disease cohort (same patients as Figure 1). (*I*) Description of biopsy locations: BF^+^ biopsies (*orange*) were taken from areas in the cecum or ascending colon with endoscopically visible biofilm, BF^–^ biopsies (*blue*) were taken in the same area of BF^–^ patients. Distal-Bx (biopsies) (*yellow*) were taken from BF^+^ patients in distal areas without endoscopically visible biofilms. (*J–R*) Same samples as for Supplementary Figure 4*A–H* with addition of data from Distal-Bx (*yellow*). (*A, J*) Total number of bacteria normalized to length of epithelium per section. (*B, K*) Maximum density of bacteria in one 144.7 × 144.7 μm confocal microscopy image per section. (*C, L*) Maximum width of methacarn-fixed H&E surface layer on top of epithelium in each section. (*D, M*) Ratio of quantitative polymerase chain reaction determined 16S rRNA gene copies to total DNA of biopsies. (*E, N*) Number of bacteria 3 μm from the epithelium, normalized to length of epithelium per section. (*F, O*) Maximum density of bacteria up to 3 μm distal from the epithelium in one 144 × 144 μm confocal microscopy image per section. (*G, P*) Maximum width of methacarn-fixed PAS surface layer on top of epithelium per section. (*H, Q*) Average width of methacarn-fixed PAS-stained surface layer on top of epithelium per section. (*R*) Percentage of biopsies with more than 10^9^ · mL^–1^ bacteria invading the mucus layer in at least one 144.7 × 144.7 μm confocal microscopy image. (*S*) Correlation matrix with Pearson correlation coefficients of microscopic data of BF^+^ biopsies and BF^–^ biopsies, *P* values ≤ .05 have been *colored*. (*T*) Location of endoscopically visible biofilms in IBS, UC, and controls. ∗*P* ≤ .05; ∗∗*P* ≤ 0.01; ∗∗∗*P* ≤ .001. (*A, B, E, F, J, K, N, O*) Zero values are displayed in direct contact with the x-axis, as they are not defined on a log-scale. (*A*–*C, E, F, J*–*L, N, O*) Kruskal-Wallis test with Dunn’s multiple comparison test, (*D, G, H, M, P, Q*) 1-way analysis of variance with Turkey’s multiple comparison test on log-transformed data, (*R*) Fisher exact test with Bonferroni correction for multiple comparisons. (*A–C, E–L, N–S*) n = 37 BF^+^, n = 47 BF^–^, n = 30 Distal-Bx (*D, M*) n = 42 BF^+^, n = 56 BF^–^, n = 35 Distal-Bx.

During diagnostic colonoscopy in patients with IBS, we frequently observed yellow-green layers that adhered to the ileal and right-colonic mucinous surface, despite proper bowel preparations with a PEG-based solution (for a macroscopic definition of biofilms see Table 1). These layers could cover several decimeters or the whole gut and would only detach upon intensive jet washing in a film-like manner (Supplementary Video 1, Figure 1*A*, and Supplementary Figure 1*A*). Investigation of several specimens under conventional bright-field microscopy and SEM revealed the presence of dense bacterial agglomerates (Figure 1*B* and Supplementary Figure 1*A–C*), indicating that these layers were bacterial biofilms. To further validate this finding, we compared colonic biopsies of BF^+^ areas to biopsies of the same area from patients without biofilms (BF^–^). We quantified the number and density of bacteria in these biopsies using U-Net,^29^ a deep learning algorithm that was trained to detect bacteria on DAPI-stained confocal microscopy images (Supplementary Figures 2*A* and 3) and identified an approximately 10-fold increase in BF^+^ compared with BF^–^ biopsies (Figure 1*D* and *E*). SEM of BF^+^ biopsies confirmed dense bacterial layers in direct contact with the epithelium, whereas BF^–^ biopsies had intact mucus layers with scattered bacteria on the mucus layer surface (Figure 1*B*, Supplementary Figure 1*B* and *C*). BF^+^ biopsies also had a higher number of bacteria adhering to the epithelium (Figure 1*F*). In 2 BF^+^ biopsies from patients with IBS, bacteria were invading the epithelium at a single location (Supplementary Figure 2*B*). To verify the higher bacterial densities observed in BF^+^ biopsies with an independent molecular approach, we quantified bacterial 16S rRNA gene copy numbers in DNA extracts obtained from biopsy samples using quantitative polymerase chain reaction. BF^+^ biopsies had significantly more bacterial DNA than BF^–^ biopsies (Figure 1*G*). The total amount of bacteria determined histologically correlated with the number of epithelial-adherent bacteria and quantitative polymerase chain reaction data, which further validated our approach (Supplementary Figure 4*S*). Differences were also evident in methacarn-fixed and H&E-stained histologic biopsy sections, with BF^+^ biopsies having a thick surface layer comprising mucus and bacteria in direct contact with the epithelium (Figure 1*C* and *H*). As additional readout for the intestinal mucus layer, we performed PAS staining. There was an increase in maximum PAS-stained layer height, but not average PAS-stained layer height in BF^+^ biopsies (Supplementary Figure 4*P* and *Q*). Taken together, we concluded that the yellow-green layers were indeed macroscopically visible biofilms that can be detected during diagnostic high-definition white light endoscopy. This phenotype was present in patients with IBS and patients with UC, as well as in some otherwise healthy individuals (Supplementary Figure 4*A–H*). We also observed a trend towards higher bacterial load, density, and adherence in biopsies taken from another more distal colonic area without such visible biofilms in BF^+^ patients (Distal-Bx; Supplementary Figure 4*I*) compared with BF^–^ patients, suggesting a colonic field effect of alterations in the microbiota (Supplementary Figure 4*J–R*). Biofilms have been defined previously as >10^9^ · mL^–1^ bacterial invasions of the mucus layer.^36^ Applying this threshold to the confocal microscopy data, 89% of BF^+^ patients (determined by our established endoscopic features for macroscopic biofilm detection) (Table 1) fulfilled such criteria in comparison with 40% of BF^*–*^ patients (Supplementary Figure 4*R*, 72% accuracy).

**Table 1:** Definition of Endoscopic Biofilms

Variable	Endoscopic appearance after high-volume PEG bowel preparation, BBPS ≥6	
	Fecal remnants	Biofilm
Location	Anywhere between cecum and rectum	Ileocecal +/– ascending colon
Morphology	Spotty	Continuous green-yellow layer, sometimes patchy
Circumferential location	Enhanced material on lower side^a^	All sides with similar pattern
Waterjet washing	Easy to wash off	Hard to wash off, comes off as membrane
Postwash remnants	No remnants using NBI	Red spots when using NBI

BBPS, Boston Bowel Preparation Scale; NBI, narrow-band imaging.

a Due to gravity.

### Mucosal Biofilms Are an Endoscopic Feature Observed Frequently in Irritable Bowel Syndrome and Ulcerative Colitis

**Supplementary Figure 5:**
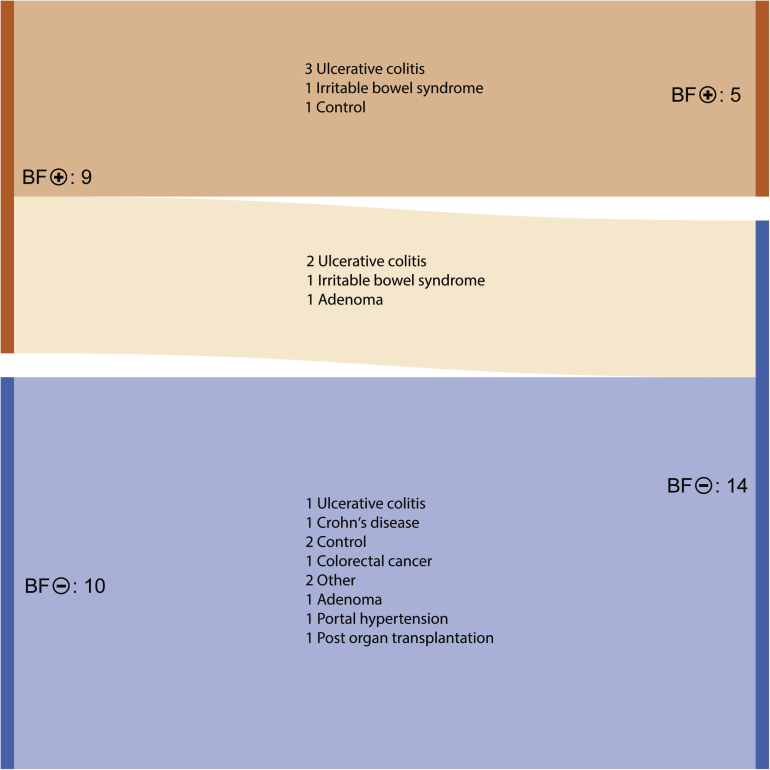
Sankey diagram of longitudinal biofilm status. Biofilm status of longitudinal patients in the primary patient cohort, first time point on the *left* and second time point on the *right side*. Patient flows have been *colored* according to biofilm status and include disease cohorts, BF^+^ on both time points (*orange*), BF^+^ on the first time point and BF^–^ on the second time point (*light orange*), and BF^–^ on both time points (*blue*). *Other* represents GI infection– and chemotherapy-induced diarrhea, n = 19 patients, average time between colonoscopies was 7 months.

The prevalence of endoscopically visible biofilms was studied in 2 independent university-based endoscopy units. All patients scheduled for an endoscopy with PEG-based bowel preparation and sufficient bowel preparation scale (Boston Bowel Preparation Scale score ≥6) were included in the study and grouped according to their underlying pathologies. Biofilm status was determined according to our established criteria (Table 1, Supplementary Video 1). We identified biofilms in the ileum and/or colon in 212 of 1112 colonoscopies (19%). Biofilms were prevalent in patients with IBS (57%), UC (34%), after organ transplantation (23%), and in patients with Crohn’s disease (22%), but not among healthy controls undergoing screening colonoscopies (6%) (Table 2). A multivariate logistic regression excluded the possibility of age and sex influencing our analysis (Supplementary Tables 1 and 2). Also, the endoscopy unit had no significant effect in the multivariate logistic-regression model (*P* = .97). When comparing the biofilm prevalence between the Austria and the German cohorts directly, the Austrian adenoma cohort (*P* = .036) and German Crohn’s disease cohort (*P* = .007) had significantly higher prevalence of biofilms. Biofilms were commonly located in the cecum (72%), terminal ileum (71%), ascending colon (45%) and, to a lesser extent, in the transverse colon (18%), descending colon (11%), sigmoid colon (8%), and rectum (6%) (Figure 1*I*). Ileal biofilms concurred with right-sided colonic biofilms in 66 of 95 cases (70%). Endoscopically visible biofilms were most prevalent in the ileocecal region, independent of pathology. There was a trend that biofilms extended further distal in patients with UC (Supplementary Figure 4*T*). Ten of 10 BF^–^ patients maintained their phenotype upon a follow-up colonoscopy. Four of 9 BF^+^ patients switched to BF^–^, with an average time between longitudinal colonoscopies of 7 months (Supplementary Figure 5). In UC, the presence of biofilms was associated with disease extent and a trend toward histologic inflammation (Supplementary Tables 1 and 3). To investigate this connection further, fecal calprotectin was analyzed as a marker for intestinal inflammation in a representative subgroup of patients. BF^+^ patients indeed had higher calprotectin values compared to BF^–^ patients. This effect was more pronounced in patients with UC, with an approximately 10-fold increase in calprotectin in BF^+^ patients (Supplementary Table 4). Medication can influence microbiome composition and intestinal mucus production.^24^^,^^37^ Therefore, we analyzed the medication history in BF^+^ and BF^–^ patients. This analysis revealed an association of proton pump inhibitors and presence of biofilms in otherwise healthy individuals (Supplementary Table 4). No association between recent antibiotics intake, probiotics, nonsteroidal anti-inflammatory drugs, or thyroid hormone therapy was found.

**Table 2:** Prevalence of Endoscopically Visible Biofilms in 2 Independent Endoscopy Units

Variable	Biofilm prevalence: BF^+^/total cases (%)			OR^a^ (95% CI)
	Total	Austria	Germany	
Irritable bowel syndrome	65/114 (57)	52/86 (60)	13/28 (46)	19.2 (9.5–42.5)∗∗∗
Ulcerative colitis	46/136 (34)	30/102 (29)	16/34 (47)	7.4 (3.7–16.2)∗∗∗
Post organ transplantation	9/39 (23)	6/28 (21)	3/11 (27)	4.3 (1.6–11.7)∗∗
Crohn’s disease	30/134 (22)	10/82 (12)	20/52 (38)	4.2 (2.0–9.3)∗∗∗
Other^b^	7/50 (14)	7/49 (14)	0/1 (0)	—
Adenoma	26/208 (13)	24/142 (17)	2/66 (3)	—
Portal hypertension	8/67 (12)	6/48 (13)	2/19 (11)	—
Colorectal cancer	4/39 (10)	4/26 (15)	0/13 (0)	—
Diverticular disease	4/92 (4)	3/29 (10)	1/63 (2)	—
GI bleeding	3/78 (4)	2/52 (4)	1/26 (4)	—
Healthy control	10/155 (6)	8/112 (7)	2/43 (5)	—
Total	212/1112 (19)	152/756 (20)	60/356 (17)	—

NSAID, nonsteroidal anti-inflammatory drugs; OR, odds ratio.

a Only significant and adjusted OR are shown.

b Including microscopic, collagen, eosinophilic, and NSAID colitis, and GI infection– and chemotherapy-induced diarrhea.

∗∗ *P* ≤ .01.

∗∗∗ *P* ≤ .001.

### Bacterial Biofilms Are Linked to a Dysbiotic Microbiome and Increased Levels of Intestinal Bile Acids

**Figure 2:**
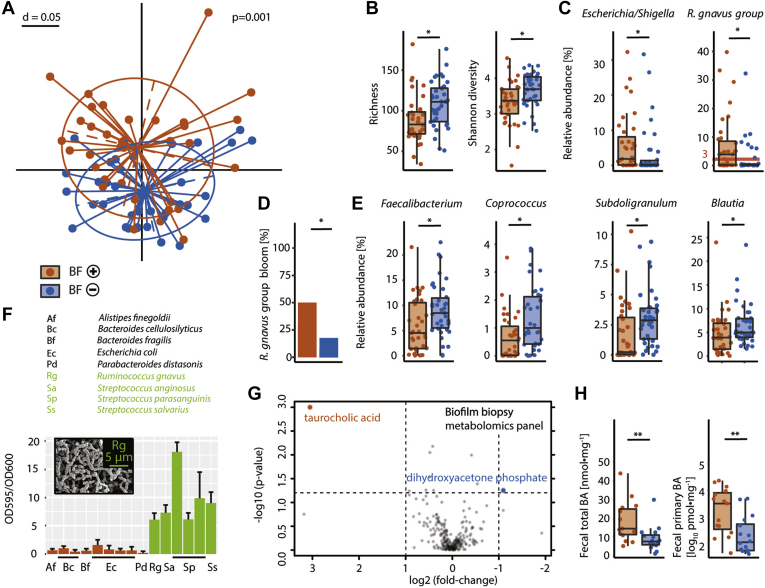
Bacterial dysbiosis, spontaneous biofilm formation, and increased BA levels in BF^+^ patients. (*A*) Multidimensional scaling plot of bacterial profiles (16S, generalized UniFrac distances) from colonic BF^+^ biopsies (*orange*) and BF^–^ biopsies (*blue*), including patients with IBS, patients with UC, and healthy controls. (*B*) BF^+^ biopsies had bacterial dysbiosis (reduced richness and Shannon diversity index). (*C*) BF^+^ biopsies were enriched in bacteria from the *Escherichia/Shigella* genus and *R. gnavu*s group. (*D*) 51% of BF^+^ biopsies had a bloom of *R. gnavus* compared with 18% of BF^–^ biopsies. (*E*) BF^+^ biopsies had a reduction in short-chain fatty acid–producing genera, including *Faecalibacterium*, *Coprococcus*, *Subdoligranulum*, and *Blautia.* (*F*) In vitro biofilm formation assay of 15 bacterial isolates from 6 BF^+^ brushes (2 controls, 4 patients with IBS). Strains with >5 OD595/OD600 ratio were defined as biofilm formers and are *green*. *Inset:* SEM picture of *R. gnavus* biofilm. (*G*) *Volcano plot* of metabolomics panel revealing an enrichment of taurocholic acid (the only BA in our metabolite panel) and a reduction of dihydroxyacetone phosphate in BF^+^ biopsies, *P* value threshold .05; log_2_ fold-change threshold ±1. (*H*) Increase of total and primary BA in stool samples from BF^+^ Patients with IBS. Statistical analysis: (*A*) Permutational multivariate analysis of variance of the distance matrices, (*B–C, E*) Kruskal-Wallis rank sum test with Benjamini-Hochberg correction for multiple comparisons, (*D*) Fisher exact test, (*H*) Mann-Whitney U test; (*A–E*) n = 35 BF^+^, n = 38 BF^–^, (*F*) n = 8 replicates per strain, (*G*) n = 5 BF^+^, n = 5 BF^*–*^, (*H*) n = 14 BF^+^, n = 14 BF^*–*^; ∗*P* ≤ .05, ∗∗*P* ≤ .01.

**Supplementary Figure 6:**
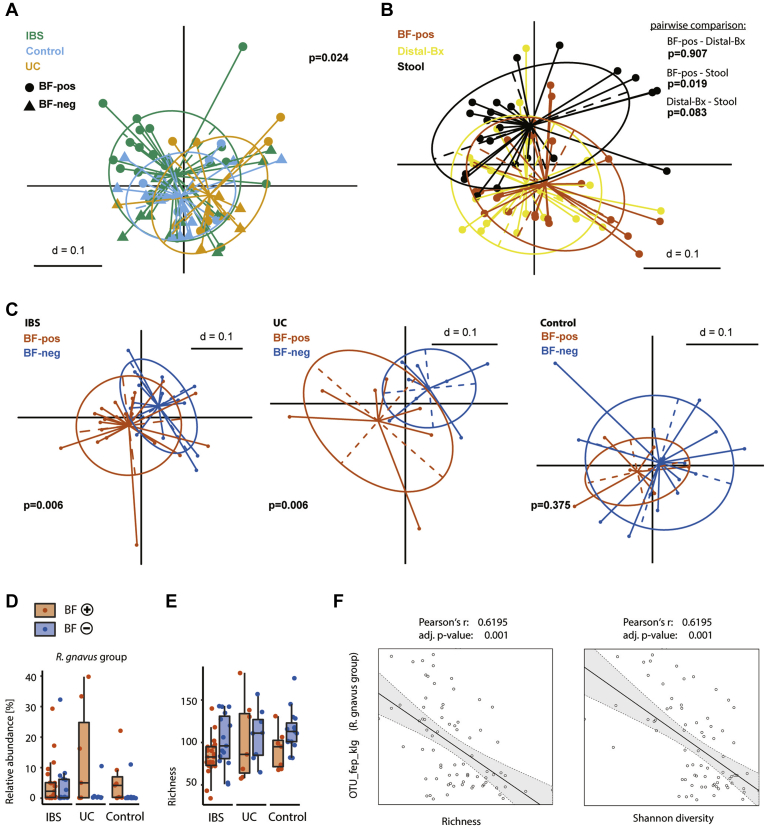
Microbial biofilm composition separated for disease. (*A*) Samples from Figure 2 with multidimensional scaling plot of generalized UniFrac distances from BF^+^ (*circle*) and BF^–^ patients (*triangle*) *colored* for disease cohort. (*B*) Intra-patient comparison of stool samples (*black*), BF^+^ biopsies (*orange*), and Distal-Bx (*yellow*). (*C*) Samples from Figure 2 split for disease cohorts. (*D*) Comparison of *R. gnavus* group relative abundance and bacterial richness between BF^+^ biopsies (*orange*) and BF^–^ biopsies (*blue*), split for disease cohort (using data from Figure 2*B* and *E*). (*F*) Correlation of colonic *R. gnavus* OTU relative abundance with richness and Shannon diversity of colonic biopsies. (*A–C*) *P* value represents a permutational multivariate analysis of variance of generalized unifrac distance matrices. (*D, E*) Kruskal-Wallis rank sum test with Benjamini-Hochberg correction for multiple comparisons. (*F*) Pearson correlation of centered log-ratio transformed OTU abundance, richness, and Shannon diversity. (*A, C–F*) n = 35 BF^+^, n = 38 BF^–^ (*B*) n = 22 BF^+^, n = 22 Distal-Bx, n = 22 BF^+^ stool samples.

**Supplementary Figure 7:**
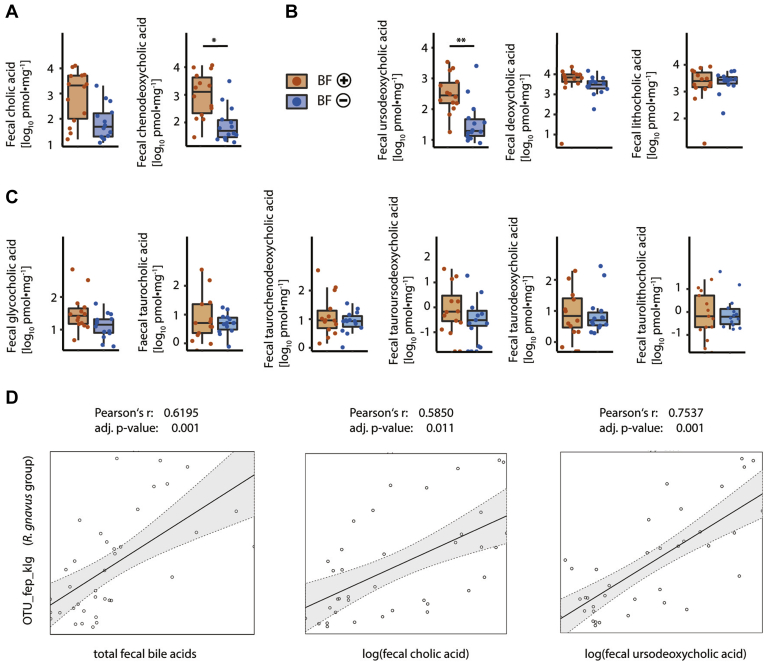
Fecal BA levels of BF^+^ and BF^–^ patients with IBS. (*A*) Levels of primary BAs in stool samples of BF^+^ (*orange*) and BF^–^ (*blue*) patients with IBS. (*B*) Levels of secondary BA in stool samples of BF^+^ and BF^–^ patients with IBS. (*C*) Levels of tauro- and glycol-conjugated BA in stool samples of BF^+^ and BF^–^ patients with IBS. (*D*) Correlation of colonic *R. gnavus* OTU relative abundance and fecal BA levels. (*A–C*) Mann Whitney U test with Bonferroni correction for multiple comparisons. (*D*) Pearson correlation of centered log-ratio transformed OTU abundance and fecal BA levels. (*A–C*) n = 14 BF^+^, n = 14 BF^–^, (*D*) n = 40.

**Supplementary Figure 8:**
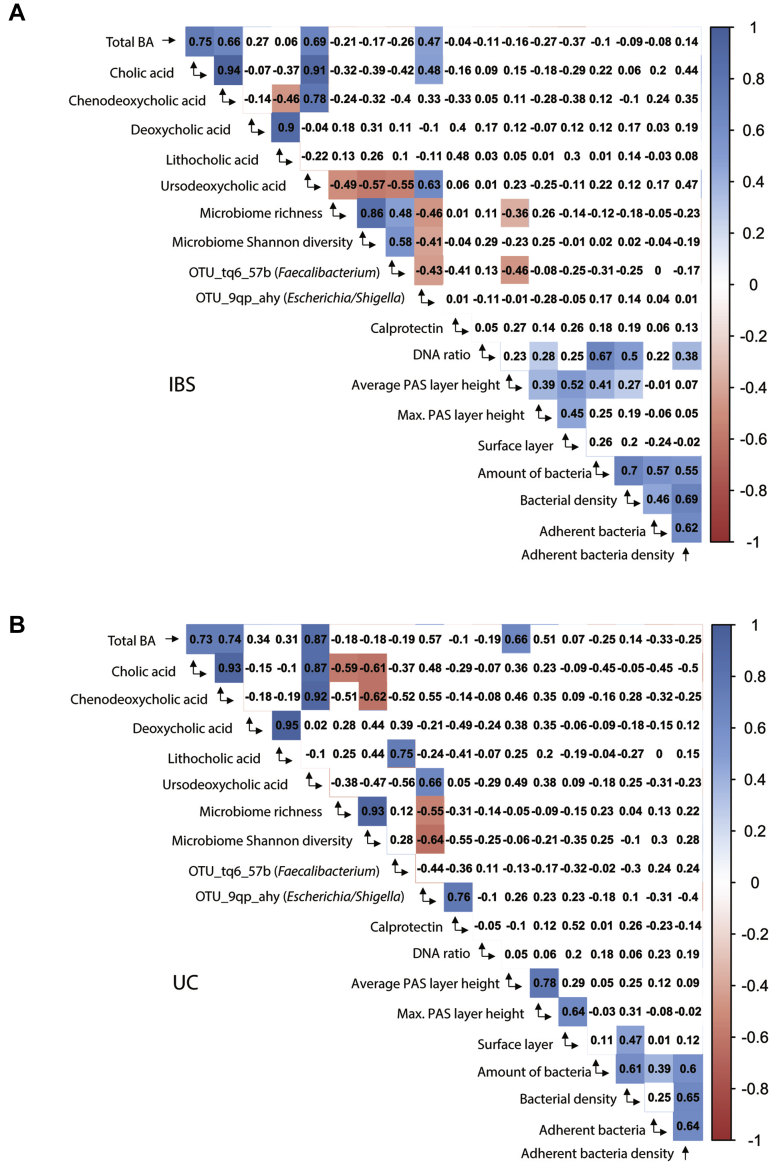
Exploratory correlation matrices of calprotectin, microbiome, microscopic, and BA data in IBS and UC. Correlation matrices with Pearson correlation coefficients for patients with IBS (*top panel*) and patients with UC (*bottom panel*). Microscopic data from colonic biopsies (average and maximum width of methacarn-fixed PAS-stained surface layer on top of epithelium per section [average PAS layer height, maximum PAS layer height], maximum width of methacarn-fixed H&E surface layer on top of epithelium per section [surface layer], total number of bacteria normalized to length of epithelium per section [amount of bacteria], maximum density of bacteria in one 144.7 × 144.7 μm confocal microscopy image per section [bacterial density], number of bacteria 3 μm from the epithelium, normalized to length of epithelium per section [adherent bacteria], maximum density of bacteria up to 3 μm distal from the epithelium in one 144 × 144 μm confocal microscopy image per section [adherent bacteria density]), ratio of quantitative polymerase chain reaction determined 16S rRNA gene copies to total DNA of biopsies (DNA ratio), microbiome data from colonic biopsies (relative abundance of OTU_tq6_57b belonging to the *Faecalibacterium* genus and OTU_9qp_ahy belonging to the *Escherichia/Shigella* genus, microbiome richness and Shannon diversity), BA data from stool samples (total BA, cholic acid, chenodeoxycholic acid, deoxycholic acid, lithocholic acid, and UCDA), calprotectin data from stool samples (calprotectin). Positive correlation coefficients in *blue*, negative correlation coefficients in *red*. *P* values ≤.05 have been *colored*. (*A*) n = 61. (*B*) n = 30.

16S rRNA gene amplicon sequencing analysis of colonic biopsies from UC, IBS, and control patients revealed a significantly altered microbiome in BF^+^ patients compared with all other samples, regardless of disease state (Figure 2*A*, Supplementary Figure 6). Overall, BF^+^ patients had a decrease in bacterial richness and diversity (Figure 2*B*). Bacteria belonging to *Escherichia*/*Shigella* genus and *Ruminococcus gnavus* group were particularly increased in BF^+^ biopsies (Figure 2*C*). Overall, 51% of BF^+^ and 18% of BF^–^ patients had a bloom of the *R. gnavus* group (Figure 2*D*). The presence of biofilms was associated with a decrease of short-chain fatty acid–producing bacterial genera, including *Faecalibacterium*, *Coprococcus*, *Subdoligranulum*, and *Blautia* (Figure 2*E*). In vitro assays of 15 representative strains isolated from brush samples collected endoscopically from 6 colonic biofilms identified 6 strong biofilm producers: 1 *R. gnavus* and 5 *Streptococci* (including 3 *Streptococcus parasanguinis*, which are known to form dental plaque). The *Escherichia coli* isolates did not spontaneously produce biofilms in vitro (Figure 2*F*). Metabolomic analysis of ileal biopsies with and without a biofilm revealed an accumulation of taurocholic acid—the only BA in our metabolite panel—in BF^+^ biopsies, together with a reduction of dihydroxyacetone phosphate (Figure 2*G*), a bacterial metabolite that inhibits biofilm formation.^38^ In addition, stool samples from patients with IBS had twice the amount of total BA and an approximately 10-fold increase of primary BA and ursodeoxycholic acid (UDCA) in BF^+^ patients compared with BF^–^ patients (Figure 2*H*, Supplementary Figure 7). The total stool BA levels, the primary BA cholic acid and UDCA correlated with a bacterial operational taxonomic unit (OTU) belonging to the *R. gnavus* group (Supplementary Figure 7*D*), which play a key role in BA metabolism.^39^ An exploratory analysis investigating the correlation between microbiome, microscopic data, calprotectin, and fecal BA was performed using Pearson correlation coefficient matrices. In IBS, microbial diversity was negatively correlated with UDCA levels and relative abundances of an OTU belonging to *Escherichia*/*Shigella* (Supplementary Figure 8*A*). An OTU belonging to *Faecalibacterium* was positively correlated with microbial diversity and negatively correlated with the OTU belonging to *Escherichia*/*Shigella* and UDCA. Average PAS layer height as a measure for intestinal mucus production was correlated with the amount and density of bacteria (Supplementary Figure 8*A*). In UC, microbiome diversity was negatively correlated with primary BA levels and relative abundances of the *Escherichia*/*Shigella* OTU. The same OTUs also correlated with intestinal inflammation, as measured by calprotectin. Average PAS layer height was correlated to the total amount of BA (Supplementary Figure 8*B*).

### Biofilms Have Disease-Specific Signatures of Bacterial Amplicon Sequencing Variants

**Figure 3:**
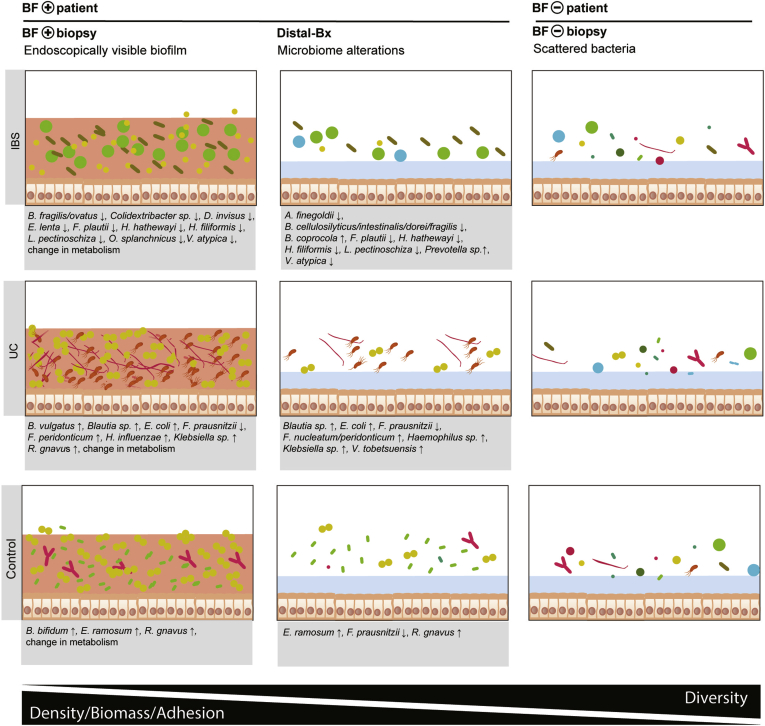
Bacterial signatures in intestinal biofilms of patients with IBS, patients with UC, and healthy controls. Changes of bacterial ASVs in BF^+^ patients at areas with (BF^+^ biopsy) and without (Distal-Bx) endoscopically visible biofilms vs BF^–^ patients (BF^–^ biopsy), for IBS, UC, and healthy controls. Mucus layer (*blue*), biofilm (*red*). For each ASV, the bacterial species or genus is listed. n = 35 BF^+^ biopsies, n = 38 BF^*–*^ biopsies, n = 30 Distal-Bx, n = 51 BF^+^ stool, and n = 54 BF^*–*^ stool samples.

**Supplementary Figure 9:**
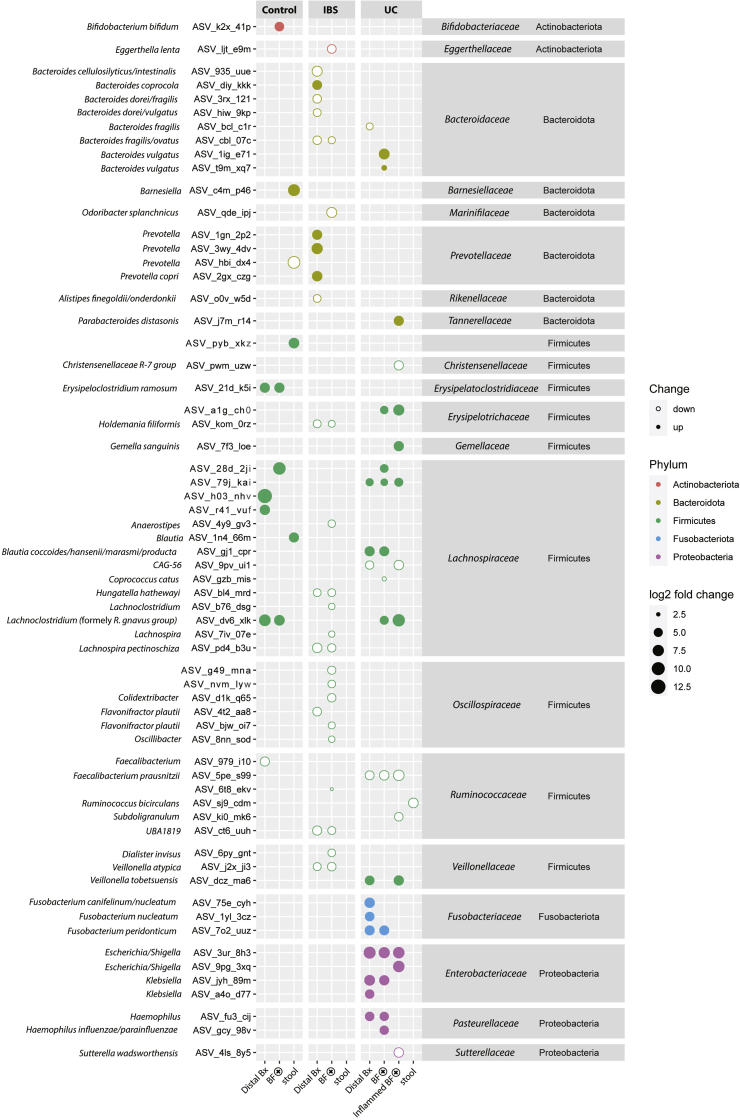
Biofilms show disease-specific changes on ASV level. ASV-based differences (as determined with DESeq2) between BF^–^ and BF^+^ biopsies, between BF^–^ and areas without biofilm from BF^+^ patients (Distal-Bx), stool from BF^+^ and BF^–^ patients, in each disease cohort. Additional comparison between UC inflamed areas of BF^+^ (inflamed BF^+^) and BF^–^ patients. Size of *dots* represent fold-change, *full dots* represent up-regulation in biofilm setting, *empty dots* represent down-regulation. *Dots* are *colored* based on bacterial phylum, bacterial taxonomies (genera and species) have been assigned to each ASV based on the SILVA reference database. Only significant findings (*P* < .05 after correction for multiple comparisons) are shown. n = 35 BF^+^, n = 38 BF^–^, n = 30 Distal-Bx, n = 5 inflamed BF^+^, n = 6 inflamed BF^–^, n = 51 BF^+^ stool samples, n = 54 BF^–^ stool samples.

To obtain a more comprehensive picture of microbiome changes in BF^+^ patients, we compared the abundances of individual DNA sequences (ASVs) of colonic BF^+^ biopsies, Distal-Bx (biopsies taken from a distal area without visible biofilms in BF^+^ patients, see Supplementary Figure 4*I*) and stool with respective samples from BF^–^ patients in different disease states. Healthy controls, patients with IBS, and patients with UC had a distinct signature of bacterial ASVs upon biofilm formation (Figure 3, Supplementary Figure 9). An ASV belonging to a *R. gnavus* strain was increased in biofilms from patients with UC and control subjects. ASVs belonging to the *Escherichia/Shigella* genus were increased in UC biofilms, further underscoring the importance of *E. coli* in IBD pathogenesis. *R. gnavus* and *Escherichia/Shigella* ASVs were also enriched in inflamed tissue of BF^+^ patients with UC compared with inflamed tissue of BF^–^ patients with UC. In addition, there was an increase of ASVs belonging to *Bacteroides vulgatus* strains and the opportunistic pathogens *Haemophilus influenzae*, *Fusobacterium*, and *Klebsiella* species in UC biofilms. IBS biofilms were depleted of several bacterial ASVs, including taxa that are considered to be commensals: *Bacteroides ovatus*, *Veillonella atypica*, *Dialister invisus*, and *Lachnospira* species. In addition to *R. gnavus*, biofilms from control subjects had increased levels of *Erysipeloclostridium ramosum* and *Bifidobacterium bifidum* ASVs (Supplementary Figure 9). In BF^+^ patients, BF^+^ biopsies and Distal-Bx were similar, as visualized by ordination of bacterial β-diversity (Supplementary Figure 6*B*). It is plausible that shed biofilm bacteria can influence the microbial composition of the whole colon. Comparing BF^–^ with Distal-Bx samples on the ASV level confirmed the overall findings of the BF^+^ vs BF^–^ biopsy analysis (Figure 3, Supplementary Figure 9). However, subtle differences between BF^+^ and Distal-Bx exist: Distal-Bx of patients with UC had an increase of an ASV belonging to *Veillonella tobetsuensis*, an early colonizer in oral biofilm formation,^26^ which was not detected in BF^+^ UC biopsies (Supplementary Figure 9). Distal-Bx samples from patients with IBS had an increase of ASVs belonging to the *Prevotella* genus and *Bacteroides coprocola*, which was not evident in BF^+^ IBS biopsies (Supplementary Figure 9). Overall, these findings support the concept that biofilm formation is a disease-specific process involving a dysbiotic microbiota in a susceptible host.

## Discussion

Biofilms provide bacteria a competitive advantage, as they protect against external stressors and enable the exchange of genetic information and nutrients.^40^^,^^41^ Unlike in the oral cavity and the appendix, the existence of biofilms in the remaining GI tract has long been a matter of debate.^14^^,^^15^ Biofilms have been observed microscopically in IBD,^42^ *H pylori* infection,^43^ and colorectal cancer.^36^^,^^44^, ^45^, ^46^ Despite their potential role in disease pathogenesis, intestinal biofilms remain understudied.^14^ Here, we present compelling evidence that intestinal biofilms are a common feature of IBS and IBD and are readily visible during endoscopy. Such biofilms have been observed by endoscopists for many years but have been misinterpreted as incomplete bowel cleansing. As biofilms are present in more than half of all patients with IBS and one-third of patients with UC, it strongly implicates their involvement in disease pathogenesis and provides a strong basis for new diagnostic and treatment opportunities, as well as disease classification (BF^+/–^).

Both participating centers detected biofilms primarily in patients with IBS, IBD, or after organ transplantation, all disease cohorts with a disturbed microbiota.^47^^,^^48^ Healthy controls had low biofilm prevalence in both centers, which supports the hypothesis that biofilms represent a pathological state of the microbiome. Besides, more patients with Crohn’s disease had biofilms in the German center, and the Austrian center had relatively more biofilms in the adenoma cohort. Such differences point to confounding factors like demographics, nutrition, and medication, which need to be examined further. We found that medications such as proton pump inhibitors can increase biofilm presence. Future studies on biofilms should also target secondary care and involve detailed questionnaires about nutritional habits and medication.

Biofilms occurred mainly in the ileum and cecum, and to a lesser extent toward the distal colon, independent of pathology. The cecum has the largest diameter of the entire intestine, has long feces retention times, and is close to the biofilm-rich appendix, which might explain biofilm formation there. IBS has been connected to a prolonged orocecal intestinal transit time, which could facilitate bacterial adhesion to ileal mucosa.^49^ In addition, the ileum and cecum harbor relatively high BA concentrations, which can trigger biofilm formation.^50^ As the present study focused exclusively on biofilms observable by colonoscopy, the prevalence of biofilms in the intestinal tract in IBS may be underestimated. Small bowel capsule endoscopy studies could reveal further prevalence of biofilms in areas of the gut that are not easily accessible via flexible endoscopy. Endoscopists participating in this study also observed biofilms in other areas of the GI tract, such as the stomach (rarely) and the upper jejunum (in patients with small intestinal bacterial overgrowth).

Microscopic biofilms were defined previously as >10^9^ bacteria · mL^–1^ invading the mucus layer.^36^ Accuracy to detect such microscopic biofilms macroscopically during endoscopy was 72%, which is comparable to endoscopic vs histologic characterization of polyps during screening colonoscopy.^51^ However, 40% of patients that met the criteria for a microscopic biofilm had no endoscopic biofilm in our analysis. This could be explained by methodological differences, as this is the first study to quantify bacteria using pattern recognition and deep learning. It might also be the case that such patients had endoscopic biofilms in areas of the GI tract that were not investigated.

Biofilms correlated with a less diverse microbiome, with overgrowth of *R. gnavus* and *E. coli*. Microbial dysbiosis has been linked to IBS,^11^ IBDs,^11^ and colorectal cancer.^52^ BF^+^ patients had reduced abundances of commensal *Faecalibacterium* and *Blautia* species, which are currently being tested in clinical trials for treatment of UC and IBS.^53^^,^^54^ Such biofilms have the potential to become visible biomarkers of disturbed microbiota homeostasis and may become a diagnostic hallmark for IBS. Because biofilms are also present in healthy individual undergoing surveillance endoscopy, they may exist without associated symptoms or be a warning signal of a tipping point^55^ between a healthy ecological equilibrium and a deregulated state that is recalcitrant to outside interventions (ie, host immune system or antibiotics) and prone to develop GI disease. Endoscopists need to be aware that such biofilms are not a matter of incomplete bowel cleansing but rather need endoscopic removal by flushing to improve visualization of the underlying mucosal surface. In addition, BF^+^ patients may need shorter surveillance intervals, as they may be at risk for development of right-colonic neoplasia.^46^

The modest sample size of the in-depth molecular and microscopic cohort in combination with complex pathologies, such as IBS and IBD, is a limitation of this study. The applied metabolomics panel only included 1 BA and lacked important metabolites as N^1^,N^12^-diacetylspermine, which has been shown to be associated with colonic biofilms.^44^ Four of 9 patients changed their biofilm phenotype from BF^+^ to BF^–^. Further large-scale longitudinal studies need to be performed to get insights into metabolic drivers of biofilm formation and connection to GI symptoms. In addition, the scoping physicians were aware of diagnosis and biofilm location, which could have led to bias. In vitro experiments, blinded scoring of biofilm status and location, and blinded intervention trials need to be done to establish causality between biofilms and GI symptoms.

The physical nature and size of these biofilms (adhesion properties, hydrophobicity, elasticity, and extent) could impair peristalsis and pose a diffusion barrier, which could contribute to or even explain common functional symptoms, such as BA-induced diarrhea, bloating, and pain. Indeed, an increase in BA was observed in both biofilms and feces of BF^+^ patients with IBS, supporting this hypothesis. A recent study also reported BA malabsorption along with increased levels of *R. gnavus* in fecal samples of patients with IBS.^7^ Biofilms might disrupt the protective mucus layer, as indicated by our SEM pictures and the observed increase in epithelium-adherent bacteria in BF^+^ biopsies. This might subsequently lead to immune system activation via *E. coli* virulence factors,^56^ *R. gnavus* inflammatory polysaccharides,^57^ and impaired barrier function by increased BA levels.^58^^,^^59^ An increase of bacteria close to the epithelium can also stimulate mucus production.^60^ *R. gnavus*, as mucus degrader and forager, might benefit from elevated mucus levels.^61^ In patients with IBS, but not patients with UC, average PAS layer height as a marker for mucus production correlated with amount and concentration of bacteria detected by confocal microscopy. BAs are known to increase mucus secretion and bacterial biofilm formation.^50^^,^^62^^,^^63^ In patients with UC, we observed a correlation of the average PAS layer height with total amount of BA. It is not unlikely that endoscopically visible biofilms result from increased bacterial biomass and extracellular matrix combined with elevated mucus production in the presence of BA.

*R. gnavus,* fecal cholic acid, and UCDA may be promising candidate biomarkers for early diagnosis, as well as targets for the treatment of IBS and UC. Biofilms themselves might offer a novel treatment target, as they could be mechanically or chemically disrupted to alleviate functional GI symptoms. It is also worth exploring whether BF^+^ patients with IBS benefit from BA-sequestrant treatment.^58^

In summary, we demonstrated that intestinal biofilms are visible by high-definition white light endoscopy and present in 57% of patients with IBS and 34% of patients with UC. Biofilms correlate with dysbiosis of the gut microbiome and BA malabsorption. Seventy-one percent of BF^+^ individuals had observable phenotypes of *R. gnavus* and/or *E. coli* overgrowth. Biofilms represent a new dimension in understanding GI health and disease and have the potential to revolutionize diagnostic algorithms and treatment approaches in functional GI disorders.

## Figures and Tables

**Supplementary Table 1:** Clinical Characteristics of the Primary Patient Cohort

Cohort variable	Total (n = 756)^a^	BF^–^ patients (n = 604)	BF^+^ patients (n = 152)	*P* value^b^
IBS				
Total n	86	34	52	
Sex, n (*% female*)	62 (72)	26 (76)	37 (71)	.63
Age, *y*, median (range)	43 (18–76)^∗∗^	42 (19–76)	44 (18–76)	.32
IBS-C, n (*%*)	7 (8)	4 (12)	3 (6)	.42
IBS-D, n (*%*)	38 (44)	14 (41)	24 (46)	.66
IBS-M, n (*%*)	41 (48)	16 (47)	25 (48)	1
UC				
Total n	102	72	30	
Sex (*% female*), n (*%*)	44 (43)^∗^	31 (43)	13 (43)	1
Age, *y*, median (range)	42 (12–83)^∗∗^	39 (12–83)	44 (17–79)	.5
Endoscopic disease activity (Mayo score), median (range)	1 (0–3)	1 (0–3)	0.5 (0–3)	.28
Proctitis (E1), n (*%*), n = 98	17 (17)	14 (21)	3 (10)	.26
Left-sided colitis (E2), n (*%*), n = 98	27 (28)	22 (21)	5 (17)	.14
Pancolitis (E3), n (*%*), n = 98	54 (55)	32 (47)	22 (73)	.009^∗∗^
Post-organ transplantation				
Total n	28	22	6	
Sex, n (*% female*)	13 (46)	12 (55)	1 (17)	.17
Age, *y*, median (range)	61 (21–88)^∗^	62 (21–88)	59 (55–69)	.92
Liver transplant, n (*%*), n = 27	7 (26)	4 (19)	3 (50)	.29
Heart transplant, n (*%*), n = 27	4 (15)	3 (14)	1 (17)	1
Kidney transplant, n (*%*), n = 27	13 (48)	10 (48)	3 (50)	1
Lung transplant, n (*%*), n = 27	4 (15)	4 (19)	0 (0)	.55
Crohn’s disease				
Total n	82	72	10	
Sex, n (*% female*)	35 (42)^∗^	28 (39)	7 (70)	.09
Age, *y*, median (range)	40 (19–93)^∗∗∗^	40 (19–93)	46 (26–63)	.66
Endoscopic activity (SES), median (range)	3 (0–15)	3 (0–15)	3 (0–15)	.87
Terminal ileum (L1), n (*%*)	18 (22)	18 (25)	0 (0)	.10
Colonic (L2), n (*%*)	18 (22)	15 (21)	3 (30)	.68
Ileocolonic (L3), n (*%*)	43 (52)	36 (50)	7 (70)	.32
Upper GI (L4), n (*%*)	3 (4)	3 (4)	0 (0)	1
Nonstricturing/nonpenetrating (B1), n (*%*)	37 (45)	33 (45)	4 (40)	1
Stricturing/penetrating (B2/B3), n (*%*)	45 (55)	39 (55)	6 (60)	1
Other^c^				
Total n	49	42	7	
Sex, n (*% female*)	22 (45)	19 (45)	3 (43)	1
Age, *y*, median (range)	60 (22–83)^∗∗^	60 (22–83)	59 (33–66)	.5
NSAID colitis, n (*%*)	2 (4)	2 (5)	0 (0)	1
Collagen colitis, n (*%*)	2 (4)	1 (2)	1 (14)	.27
Microscopic colitis, n (%)	2 (4)	2 (5)	0 (0)	1
GI infection, n (*%*)	15 (30)	15 (36)	0 (0)	.08
Chemotherapy-induced diarrhea, n (*%*)	28 (58)	22 (52)	6 (86)	.21
Adenoma				
Total number, n	142	118	24	
Sex, n (*% female*)	64 (45)^∗^	53 (45)	11 (46)	1
Age, *y*, median (range)	65 (23–91)^∗∗∗^	65 (23–91)	66 (30–80)	.91
Portal hypertension				
Total number, n	48	42	6	
Sex, n (*% female*)	11 (23)^∗∗∗^	9 (21)	2 (33)	.61
Age, *y*, median (range)	58 (18–83)^∗^	58 (18–83)	53 (30–66)	.13
Liver disease severity (MELD), median (range)	15 (4–40)	15 (4–40)	12 (6–20)	.16
Colorectal cancer				
Total n	26	22	4	
Sex, n (*% female*)	16 (62)	13 (60)	3 (75)	1
Age, *y*, median (range)	65 (35–79)^∗∗∗^	63 (35–79)	73 (51–79)	.34
Diverticular disease				
Total n	29	26	3	
Sex, n (*% female*)	12 (41)	10 (38)	2 (67)	.55
Age, *y*, median (range)	67 (48–87)^∗∗∗^	67 (48–87)	75 (60–75)	.5
GI bleeding				
Total n	52	50	2	
Sex, n (*% female*)	25 (48)	24 (48)	1 (50)	1
Age, *y*, median (range)	69 (16–88)^∗∗∗^	69 (16–88)	67 (61–73)	1
Healthy control				
Total n	112	104	8	
Sex, n (*% female*)	65 (59)	60 (58)	5 (63)	1
Age, *y*, median (range)	51 (19–81)	52 (19–81)	45 (22–72)	.14

NOTE. Values are presented as median (range) for continuous variables and n (*%*) for categorical variables. Percentages are calculated based on the actual number of patients in each group where the respective data were available. If data were not available for all subjects, the number of subjects for which the respective data were available is indicated in the Cohort variable column. Mann-Whitney U test and 2-sided Fisher exact test were used to determine *P* values for continuous and categorical variables, respectively.

IBS-C, irritable bowel syndrome with constipation predominant; IBS-D, irritable bowel syndrome with diarrhea predominant; IBS-M, irritable bowel syndrome with mixed bowel habits; MELD, Model for End-Stage Liver Disease; SES, Simple Endoscopic Score.

a Column includes statistical test for age and sex difference to healthy controls, ∗*P* ≤ .05; ∗∗*P* ≤ .01.

b BF^+^ patients vs BF^–^ patients, ∗∗*P* ≤ .01.

c Other represents microscopic/collagen/eosinophilic/NSAID colitis, GI infection– and chemotherapy-induced diarrhea.

**Supplementary Table 2:** Multivariate Logistic Regression of Biofilm Status in the Primary Cohort (n = 756)

Multivariate logistic regression	Crude OR (95% CI)	Adjusted OR (95% CI)	*P* value
Age	0.99 (0.98–0.99)	1.00 (0.99–1.01)	.55
Female sex	1.35 (0.94–1.92)	1.04 (0.69–1.56)	.83
Irritable bowel syndrome (n = 86)	19.88 (8.59–46.01)	20.34 (8.72–47.42)	3.1 · 10^–12^^a^
UC (n = 102)	5.42 (2.35–12.49)	5.58 (2.41–12.94)	6.2 · 10^–5^^a^
Post-organ transplantation (n = 28)	3.55 (1.12–11.24)	3.47 (1.09–11.05)	.04^b^
Adenoma (n = 142)	2.64 (1.14–6.14)	2.54 (1.08–5.98)	.03^b^
Colorectal cancer (n = 26)	2.46 (0.65–8.55)	2.25 (0.62–8.22)	.22
Other^c^ (n = 49)	2.17 (0.74–6.35)	2.12 (0.72–6.25)	.17
Crohn’s disease (n = 82)	1.81 (0.68–4.8)	1.87 (0.7–4.99)	.21
Portal hypertension (n = 48)	1.86 (0.61–5.68)	1.85 (0.6–5.72)	.29
Diverticular disease (n = 29)	1.39 (0.35–5.6)	1.33 (0.33–5.41)	.69
GI bleeding (n = 52)	0.54 (0.11–2.65)	0.51 (0.1–2.54)	.41

CI, confidence interval; OR, odds ratio.

a *P* ≤ .001.

b *P* ≤ .05.

c Representing microscopic, collagen, eosinophilic, and nonsteroidal anti-inflammatory drug–induced colitis, and GI infection– and chemotherapy-induced diarrhea.

**Supplementary Table 3:** Histopathological Assessment of Biofilm-Positive, Biofilm-Negative, and Distal Colonic Biopsies

Cohort/histopathological feature	Total (n = 109)	BF^–^ biopsies (n = 43)	BF^+^ biopsies (n = 35)	Distal-Bx (n = 31)
IBS				
Total n	50	18	19	13
Surface epithelium destruction, n (*%*)	0 (0)	0 (0)	0 (0)	0 (0)
Inflammatory infiltrate, n (*%*)	0 (0)	0 (0)	0 (0)	0 (0)
Architecture distortion, n (*%*)	0 (0)	0 (0)	0 (0)	0 (0)
UC				
Total n	30	7	12	11
Surface epithelium destruction, n (*%*)	1 (3)	0 (0)	1 (8)	0 (0)
Inflammatory infiltrate, n (*%*)	6 (20)	0 (0)	3 (25)	3 (27)
Architecture distortion, n (*%*)	7 (23)	0 (0)	5 (42)	2 (18)
Control				
Total n	29	18	4	7
Surface epithelium destruction, n (*%*)	0 (0)	0 (0)	0 (0)	0 (0)
Inflammatory infiltrate, n (*%*)	0 (0)	0 (0)	0 (0)	0 (0)
Architecture distortion, n (*%*)	0 (0)	0 (0)	0 (0)	0 (0)

**Supplementary Table 4:** Medication History and Calprotectin Data in a Subgroup of the In-Depth Molecular and Microscopic Cohort

Cohort/variable	BF^–^ patients	BF^+^ patients	*P* value^a^
Total			
Total n	58	59	—
Antibiotics, n (*%*) (n = 107)	31 (58)	33 (61)	.84
Probiotics, n (*%*) (n = 107)	7 (13)	13 (24)	.42
PPI, n (*%*) (n = 101)	13 (28)	24 (44)	.09
NSAIDs, n (*%*) (n = 107)	12 (24)	11 (20)	.64
Thyroid hormone therapy, n (*%*) (n = 113)	12 (22)	16 (28)	.52
Calprotectin, *mg/kg*, median (range) (n = 90)	1 (0–261)	30 (0–1712)	.03^∗^
UC			
Total n	9	16	—
Antibiotics, n (*%*) (n = 22)	5 (55)	8 (61)	1
Probiotics, n (*%*) (n = 24)	2 (33)	7 (47)	.67
PPI, n (*%*) (n = 24)	1 (11)	6 (40)	.19
NSAIDs, n (*%*) (n = 24)	2 (22)	4 (27)	1
Thyroid hormone therapy, n (*%*) (n = 24)	1 (11)	3 (20)	1
Calprotectin, *mg/kg*, median (range) (n = 18)	29 (0–246)	243 (0–1592)	.002^∗∗^
Control			
Total n	25	11	—
Antibiotics, n (*%*) (n = 30)	9 (43)	3 (33)	.71
Probiotics, n (*%*) (n = 33)	2 (9)	0 (0)	.54
PPI, n (*%*) (n = 31)	4 (19)	6 (60)	.04^∗^
NSAIDs (n (*%*), n = 32)	4 (19)	1 (9)	.63
Thyroid hormone therapy, n (*%*) (n = 34)	6 (26)	2 (18)	.69
Calprotectin, *mg/kg*, median (range) (n = 30)	0 (0–142)	7 (0–96)	.84
IBS			
Total n	24	32	—
Antibiotics, n (*%*) (n = 55)	17 (74)	22 (69)	.8
Probiotics, n (*%*) (n = 50)	2 (9)	6 (21)	.43
PPI, n (*%*) (n = 46)	8 (47)	12 (41)	.76
NSAIDs, n (*%*) (n = 51)	6 (28)	6 (20)	.52
Thyroid hormone therapy, n (*%*) (n = 55)	5 (22)	11 (34)	.38
Calprotectin, *mg/kg*, median (range) (n = 42)	8 (0–261)	29 (0–1712)	.2

NOTE. Values are presented as median (range) for continuous variables (calprotectin, *mg/kg* stool) or n (%) for categorical variables (antibiotic use in the last 5 years [antibiotics], probiotic use in the last 3 months [probiotics], PPI usage in the last 5 years [PPI], more than 1 NSAID per month on average in the last 3 years [NSAIDs], and thyroid hormone therapy in the last 5 years [thyroid hormone therapy]). Percentages are calculated based on the actual number of patients in each group for which the respective data were available. If data were not available for all subjects, the number of subjects for which the respective data were available is indicated in the Cohort/Variable column. Mann-Whitney U test and 2-sided Fisher exact test were used to determine *P* values for continuous and categorical variables, respectively.

NSAID, nonsteroidal anti-inflammatory drug; PPI, proton pump inhibitor.

a BF^+^ patients vs BF^–^ patients, ∗*P* ≤ .05; ∗∗*P* ≤ .01.

**Supplementary Table 5:** Patient Characteristics for Microbiome Group Comparison

Characteristic	BF^–^ biopsies	BF^+^ biopsies	*P* value^a^
Total no.	38	35	—
Sex, *female*, n (*%*)	22 (58)	22 (63)	.81
Age, *y*, median (range)	50 (19–67)	47 (17–79)	.57
Control, n (*%*)	15 (39)	7 (20)	.08
IBS, n (*%*)	15 (39)	21 (60)	.1
UC, n (*%*)	8 (22)	7 (20)	1
Antibiotic treatment in last 3 y, n (*%*) (n = 58)	13 (43)	12 (42)	1

NOTE. Values are presented as median (range) for continuous variables or n (%) for categorical variables. Percentages are calculated based on the actual number of patients in each group for which the respective data were available. If data were not available for all subjects, the number of subjects for which the respective data were available is indicated in the first column. Mann-Whitney U test and 2-sided Fisher exact test were used to determine *P* values for continuous and categorical variables, respectively.

a BF^+^ patients vs BF^–^ patients.

## Supplementary Methods

### Screening for Endoscopically Visible Biofilms

Every patient was assigned to a single hierarchically grouped disease cohort. Cohorts were grouped in the following order: post organ transplantation, colorectal cancer, portal hypertension, IBD, IBS, adenoma, GI bleeding, diverticular disease, other (including microscopic, collagen, eosinophilic, and nonsteroidal anti-inflammatory drug–induced colitis; GI infection; and chemotherapy-induced diarrhea), and healthy controls. In addition, a more detailed multivariate logistic regression with biofilm status as a dependent variable and age, sex, and disease as independent variables was calculated for the Austrian cohort. To detect country-specific differences, we compared biofilm prevalence between Austria and Germany for each disease cohort with Fisher exact test with Bonferroni correction for multiple comparisons.

### Sample Collection

Biopsies were snap-frozen in liquid nitrogen immediately and stored at –80°C until DNA extraction, or fixed in methacarn (60% methanol, 30% chloroform, and 10% glacial acetic acid) for histologic analysis, or fixed in Karnovsky's fixative (2% paraformaldehyde, 2.5% glutaraldehyde in 0.1 M phosphate buffer [pH 7.4]; Morphisto, Frankfurt am Main, Germany) for SEM. Patients sampled the first stool they produced after starting bowel preparation using a feces catcher (abbexa) and stored the stool sample at 4°C overnight. Thereafter, stool samples were aliquoted and frozen at –80°C.

### Histologic Analysis of Biofilms

Colonic biopsies were fixed in methacarn solution for 2 hours at 4°C, washed 3 times in 70% ethanol, and processed for paraffin embedding. Paraffin blocks were stored at 4°C. Colonic biopsy sections 4-μm-thick were H&E-stained or processed for fluorescence in situ hybridization (FISH) and DAPI staining. FISH staining with a 16S rRNA-targeted DNA oligonucleotide probe mix (EUB338 I-III) and confocal fluorescence microscopy imaging was performed as described previously.^1^ To visualize DNA, samples were incubated with 30 μL of DAPI (0.1 μg/mL phosphate-buffered saline) for 10 minutes in the dark.

Maximum thickness of methacarn-fixed surface layer, composed of mucus and bacteria, was assessed on H&E- and PAS-stained sections. Thickness was measured as perpendicular distance from the epithelial border to the maximum extent of the layer. Average PAS layer thickness was calculated from 3 random fields of view.

We used DAPI for deep learning–based bacterial detection because we did not achieve a constant FISH signal across all samples. This might be caused by the effect of reduced growth rate due to biofilm formation and patient medication on number and accessibility of bacterial ribosomes, which would lead to a biased analysis.^2^^,^^3^

U-Net is a recently published deep learning solution for cell quantification and annotation for the ImageJ platform (version 1.52b).^4^ We trained U-Net to detect bacteria in confocal microscopy images of DAPI-stained biopsy sections (as FISH staining did not achieve 100% hybridization efficiency). Bacteria in 14 confocal microscopy images (144.72 × 144.72 μm) were annotated through the consensus of 3 experts with a total of 5010 data points. The standard U-Net cell detection model (2d_cell_net_v0_model) was fine-tuned on our dataset with 3 images kept as reference to determine detection rates. After 10,000 iterations the algorithm reached a detection rate of 80%, which is in the range of variation of 2 humans annotating the same image (data not shown). For each bacterial count, the entire intestinal biopsy section was analyzed. Confocal microscopy images with detectable bacteria were annotated by our trained U-Net model in ImageJ. As the algorithm displayed high numbers of false negatives in areas where bacteria were in direct contact with each other, all annotations were subjected to human quality control. Maximum numbers of bacteria in each confocal microscopy image were determined and bacterial densities (per mL) calculated. Total number of bacteria was calculated as the sum of all images for each biopsy and normalized to the epithelium length (to adjust for size of biopsy sections, as determined on neighboring H&E-stained sections), resulting in number of bacteria. Adherent bacteria and adherent bacteria density were determined on confocal microscopy images as described above, but only bacteria within 3 μm from the epithelium were counted. Mann-Whitney U test was performed on nontransformed data to keep zero values. For multiple comparisons, Kruskal-Wallis test with Dunn’s multiple comparison test was applied. Data are presented on a logarithmic y-axis with base 10 and (nondefined) zero values are presented on the x-axis. Accuracy was calculated using the following formula: accuracy = (true positive + true negative) / (true positive + true negative + false positive + false negative).

### Scanning Electron Microscopy

Biopsies were immediately fixed in Karnovsky's fixative and carefully dehydrated in a graded ethanol series for SEM. Ethanol dehydration was followed by chemical drying with hexamethyldisilazane (Sigma-Aldrich). Specimens were immersed in hexamethyldisilazane for 30 minutes and air-dried. After complete evaporation of hexamethyldisilazane, samples were fixed to specimen mounts, gold sputtered (Sputter Coater Leica EM ACE200; Leica Microsystems, Wetzlar, Germany), and then examined in a scanning electron microscope (JSM 6310; Jeol Ltd, Tokyo, Japan) at an acceleration voltage of 15 kV.

### Analysis of Bacterial Community Composition

Patient characteristics and sample size, used for 16S rRNA gene amplicon sequencing analysis, are displayed in Supplementary Table 5 and Supplementary Figure 3. The DNA of colonic biopsies and stool samples was extracted using the standard QIAamp DNA stool mini kit protocol (Qiagen) modified by an initial bead-beating step with Lysing Matrix E tubes (MP Biomedicals) and a Precellys 24 homogenizer (Bertin instruments) with 5200 rpm 3 × 30 seconds for colonic biopsies and 5500 rpm 1 × 30 seconds for stool samples. Amplicon sequencing of the V3-V4 variable region of the 16S rRNA gene was performed using an established barcoding approach with 25 cycles for the first and 5 cycles for the second polymerase chain reaction and Illumina MiSeq technology (805R 5′-GACTACHVGGGTATCTAATCC-3′, 341F 5′-CCTACGGGNGGCWGCAG-3′).5 Sequencing was performed at the Joint Microbiome Facility of the Medical University of Vienna and the University of Vienna (project ID JMF-1901-5 and JMF-1910-2). ASVs were inferred using the DADA2 R ^6^package applying the recommended workflow (https://f1000research.com/articles/5-1492). FASTQ reads 1 and 2 were trimmed at 230 nt with allowed expected errors adjust per sequencing run. ASV sequences were subsequently classified using DADA2 and SINA, version 1.6.1,^7^ with the SILVA database SSU Ref NR 99 release 138^8^ using default parameters. Contaminations were identified by inspection of taxa in the negative controls compared with the true samples and removed based on taxonomy (all ASVs classified as Eukaryota, Chloroplast, Mitochondria, Bacillaceae, Oxalobacteraceae, Comamonadaceae, and Burkholderiaceae were removed). For the analysis of sample similarity modified Rhea scripts were used.^9^ Samples with fewer than 1000 reads were excluded from analysis. In case of longitudinal sampling, only the first sample from each patient was used for analysis to prevent bias. Generalized UniFrac distances^10^ were visualized using multidimensional scaling plots. Cluster significance was assessed using permutational multivariate analysis of variance. Testing for differences in diversity and bacterial abundances was performed using Kruskal-Wallis rank sum test using Benjamin-Hochberg method for multiple comparisons correction. Bacterial genera with <0.5% abundance were excluded in each sample. In addition, bacterial genera that were present in <30% of the samples were excluded to improve power. Bloom of *E. coli* and *R. gnavus* was defined as >3% relative abundance in colonic biopsies. For detailed analysis of ASVs, only samples between 2500 and 25,000 reads were included, to keep read number within 1 order of magnitude. Pairwise comparisons were performed using DESeq2^11^ between colonic BF^–^ and BF^+^ biopsies, between BF^–^ and areas without biofilm from BF^+^ patients (Distal-Bx), stool from BF^+^ and BF^–^ patients, in each disease cohort. Additional comparison between inflamed areas with biofilms of BF^+^ patients with UC (inflamed BF^+^) and inflamed areas of BF^–^ patients with UC was performed. For each comparison, samples with the least read numbers in the group with higher sample numbers were removed to achieve identical group sizes. ASVs, which were present in <25% of the samples, were removed to improve detection power. Correlations were calculated using OTUs clustered with a 94.5% sequence identity threshold. OTUs were log-ratio transformed and OTUs that were present in <30% of the samples were excluded from analysis. Zero values were treated as missing values. The table consisting of OTU and metadata was centered and scaled and Pearson correlation was calculated with Benjamini Hochberg *P* value correction.

### Quantification of Bacterial DNA

Bacterial 16S DNA was quantified using 0.2 μM 341F (5′-CCTACGGGAGGCAGCAG-3′) and 534R (5′ATTACCGCGGCTGCTGGCA -3′) universal 16S rRNA gene primers, 10 μL of 2X SYBR FAST Mastermix (Thermofisher), and 2 μL of sample DNA in 20 μL reactions. Quantitative polymerase chain reactions were run on a 7500 Fast Real-Time PCR system (Applied Biosystems, Thermo Fisher Scientific). Total amount of DNA was assessed with a Quant-iT PicoGreen dsDNA Assay Kit using standard protocols and 2 μL of sample DNA as input. Ratios of bacterial 16S rRNA gene copies/total double-stranded DNA were calculated and data were log-transformed and compared using a *t* test. Normality of log-transformed data was confirmed using D'Agostino and Pearson omnibus normality test (α = .05). For multiple comparisons, 1-way analysis of variance with Turkey’s multiple comparison test was performed on log-transformed data. Sample size was 42 BF^+^ (23 patients with IBS, 10 patients with UC, and 9 controls), 35 Distal-Bx (16 patients with IBS, 13 patients with UC, and 6 controls), and 56 BF^–^ (23 patients with IBS, 8 patients with UC, and 26 controls).

### Bile Acid Analysis of Stool Samples

Stool samples were dried in a vacuum centrifuge. Ten milligrams of dried stool was weighted into a Lysing Matrix E tube and subjected to bead-beating at 6000 rpm for 3 × 30 seconds. After homogenization, 0.4 mL of 50 mM sodium acetate (pH 5.6) and 1.1 mL acetonitrile were added to the samples and they were put for 1 hour in a heating block at 60°C. Samples were centrifuged and supernatant was taken for quantification of BAs. BAs and their conjugates were quantified using liquid chromatography–tandem mass spectrometry, employing selected reaction monitoring. In brief, 1 μL of the extract was injected on a Kinetex C_8_ column (100 Å, 100 × 2.1 mm) with the respective guard column, employing a flow rate of 100 μL/min. A 16-minute-long gradient from 80% A (2.5 mM ammonium acetate in water) to 90% B (2.5 mM ammonium acetate in methanol) was used for the separation. The high-performance liquid chromatography (RSLC ultimate 3000; Thermo Fisher Scientific) was directly coupled to a TSQ Quantiva mass spectrometer (Thermo Fisher Scientific) via electrospray ionization. BAs and their conjugates were analyzed in the negative ion mode, employing the respective transitions (eg, glycocholic acid m/z 464 to m/z 73) and an optimized collision energy, which has been determined by analyzing authentic standards. Chromatograms have been manually interpreted using trace finder (Thermo Fisher Scientific), validating experimental retention times with the respective quality controls of the pure substances. Mann-Whitney U test was performed to test for significant differences with Bonferroni correction for multiple comparisons.

### Bacterial Strain Isolation and In Vitro Bacterial Biofilm Formation Assay

Biofilm samples were collected by endoscopic brushes and cultivated on selective and nonselective media under different atmospheric conditions using established methods.^12^ Bacterial isolates were identified by matrix-assisted laser desorption/ionization time-of-flight mass spectrometry (Biotyper; Bruker Daltonics). In vitro biofilm formation experiments using standard protocols^13^ were conducted in an anaerobic tent to provide physiological conditions. Bacterial isolates were inoculated in brain heart infusion medium with supplements (37 g/L brain heart infusion, 5 g/L yeast extract, 1 g/L NaHCO_3_, 1 g/L L-cysteine, 1 mg/L vitamin K1, and 5 mg/L hemin). After 24 hours, bacterial suspensions were diluted to OD600 = 0.05 and 100 μL/well were transferred in U-bottom polystyrene 96-well plates (Costar) in 8 technical replicates. Plates were incubated at 37°C for 48 hours. Supernatants with planktonic cells were carefully removed, transferred to a new 96-well plate, and OD600 of the planktonic cells was measured. Bacterial biofilms were fixed with 150 μL BOUIN solution (0.9% picric acid, 9% formaldehyde, and 5% acetic acid) for 15 minutes and washed 3 times with 190 μL phosphate-buffered saline. For staining, 150 μL 1% crystal violet solution was added for 10 minutes and washed 3 times with 190 μL phosphate-buffered saline. For biofilm quantification, crystal violet in dried plates was dissolved in 190 μL 30% acetic acid and the plate was placed on a shaker for 1 hour. Absorbance of 1:5 dilutions was measured on a TECAN infinite 200 Pro plate reader at 595 nm and 405 nm reference wavelength. Crystal violet absorbance was multiplied by 5 to account for dilution, OD595/OD600 ratios were calculated and data are presented as averages with SDs.

### Metabolomic Analysis

Five ileal BF^+^ biopsies and 5 ileal BF^–^ biopsies were homogenized using a Precellys 24 tissue homogenizer (Precellys CK14 lysing kit; Bertin). To each milligram of tissue, 9 μL of methanol were added. Ten microliters of the homogenized tissue sample was transferred into a glass vial, 10 μL of lipid internal standard solution (SPLASH Lipidomix; Avanti Polar Lipids), 40 μL of metabolite internal standard mix, and 130 μL methanol were added. After vortexing, 500 μL methyl tert butyl ether was added and the mixture was incubated on a shaker for 10 minutes at 25°C. A phase separation was induced by adding 110 μL MS-grade water. After 10 minutes of incubation at 25°C, the samples were centrifuged at 1000*g* for 10 minutes. An aliquot of 450 μL of the upper phase (organic) was collected and dried using a nitrogen evaporator. Samples were reconstituted in 20 μL methanol and used for lipid analysis. Two hundred microliters of the lower phase (aqueous) was collected and evaporated using a nitrogen evaporator, reconstituted in 20 μL of water and used for metabolite analysis.

The liquid chromatography–mass spectrometry analysis of lipids was performed using a Vanquish UHPLC system (Thermo Fisher Scientific) combined with an Orbitrap Fusion Lumos Tribrid mass spectrometer (Thermo Fisher Scientific). Lipid separation was performed by reverse-phase chromatography employing an Accucore C_18_, 2.6 μm, 150 × 2 mm (Thermo Fisher Scientific) analytical column at a column temperature of 35°C. As the mobile phase A, an acetonitrile/water (50/50, v/v) solution containing 10 mM ammonium formate and 0.1% formic acid was used. The mobile phase B consisted of acetonitrile/isopropanol/water (10/88/2, v/v/v) containing 10 mM ammonium formate and 0.1% formic acid. The flow rate was 400 μL/min. A gradient of B was applied to ensure optimal separation of the analyzed lipid species. The mass spectrometer was operated in electron spray ionization–positive and –negative mode, capillary voltage 3500 V (positive) and 3000 V (negative), vaporize temperature 320°C, ion transfer tube temperature 285°C, sheath gas 60 arbitrary units, auxiliary gas 20 arbitrary units, and sweep gas 1 arbitrary unit. The Orbitrap MS scan mode at 120,000 mass resolution was employed for lipid detection. The scan range was set to 250–1200 m/z for both positive and negative ionization mode, the automatic gain control target was set to 2.0 × 10^5^ and the intensity threshold to 5.0 × 10^3^. The data analysis was performed using TraceFinder software (Thermo Fisher Scientific).

A 1290 Infinity II UHPLC system (Agilent Technologies) coupled with a 6470 triple quadrupole mass spectrometer (Agilent Technologies) was used for the liquid chromatography–tandem mass spectrometry analysis of metabolites. The chromatographic separation for samples was carried out on a ZORBAX RRHD Extend-C_18_, 2.1 × 150 mm, 1.8 μm analytical column (Agilent Technologies). The column was maintained at a temperature of 40°C and 4 μL of sample was injected per run. The mobile phase A was 3% methanol (v/v), 10 mM tributylamine, 15 mM acetic acid in water and the mobile phase B was 10 mM tributylamine, 15 mM acetic acid in methanol. The gradient elution with a flow rate 0.25 mL min^–1^ was performed for a total time of 24 minutes. Column was backflushed using a 6-port/2-position divert valve for 8 minutes using acetonitrile, followed by 8 minutes of column equilibration with 100% A. The triple quadrupole mass spectrometer was operated in an electrospray ionization negative mode, spray voltage 2 kV, gas temperature 150°C, gas flow 1.3 L/min, nebulizer 45 psi, sheath gas temperature 325°C, and sheath gas flow 12 L/min. The metabolites of interest were detected using a dynamic MRM mode. The MassHunter 10.0 software (Agilent Technologies) was used for the data processing. Ten-point linear calibration curves with internal standardization and was constructed for the quantification of metabolites.

Statistical analysis was performed using the MetaboAnalyst pipeline with standard settings (features with >50% missing values removed, missing values replaced with half of minimum positive value in original data, data filtering based on interquartile range, normalization by median, and log-transformation) and a threshold of .05 for *P* value and ±1 log_2_ fold-change in the volcano plot.^14^

### Calprotectin Analysis

Fecal calprotectin was analyzed using a validated commercial enzyme-linked immunosorbent assay (BÜHLMANN fCAL ELISA, BUHLMANN Diagnostics).

### Exploratory Correlation Analysis

Microscopic data, fecal BA data, quantitative polymerase chain reaction, and microbiome data from colonic biopsies were combined for each patient and log-transformed. If there were 2 biopsies from the same patient (ie, BF^+^ and Distal-Bx), only the BF^+^ biopsy was used for correlation with BA, quantitative polymerase chain reaction, and microbiome data to prevent bias. Matrices of Pearson correlation coefficients were calculated using R and the corrplot package.^15^
